# Examining discordance in spirometry reference equations: A retrospective study

**DOI:** 10.14814/phy2.70212

**Published:** 2025-02-26

**Authors:** Gerald S. Zavorsky, Sherif Elkinany, Abdullah Alismail, Suman B. Thapamagar, Michael H. Terry, James D. Anholm, Paresh C. Giri

**Affiliations:** ^1^ Department of Physiology and Membrane Biology University of California Davis California USA; ^2^ Department of Cardiopulmonary Sciences Loma Linda University Loma Linda California USA; ^3^ Department of Medicine, School of Medicine Loma Linda University Loma Linda California USA; ^4^ Department of Respiratory Care Loma Linda University Medical Center Loma Linda California USA; ^5^ Division of Pulmonary, Critical Care, Hyperbaric, and Sleep Medicine Lima Linda University Veterans Administration Loma Linda California USA; ^6^ Beaver Medical Group (Optum) Redlands California USA

**Keywords:** accuracy, ethnicity, lung function, prediction equations, pulmonary, race, reference equations

## Abstract

This study aimed to evaluate discordance, binary classification, and model fit between race‐predicted and race‐neutral spirometry prediction equations. Spirometry data from 9506 patients (18–95 years old) self‐identifying as White, Black, or Hispanic were analyzed, focusing on the lower limit of normal (LLN). Best‐fit prediction equations were developed from 3771 patients with normal spirometry, using Bayesian Information Criterion (BIC) to compare models with and without race as a covariate. Results showed that including race as a covariate improved model fit, reducing BIC by at least ten units compared to Race‐Neutral equations. Discordance between race‐specific and race‐neutral equations for detecting airway obstruction and restrictive spirometry patterns ranged from 4% to 13%. Using race‐neutral equations resulted in false discovery rates (FDR) of 14% for Hispanics and 45% for Blacks and false negative rates (FNR) of 21% for Hispanics and 27% for Blacks in diagnosing airway obstruction. These findings indicate that removing race as a covariate in spirometry equations increases FDR and FNR, leading to higher misclassification rates. The 4%–13% discordance in interpreting airway obstruction and restrictive patterns has significant clinical implications, underscoring the need for careful consideration in developing spirometry reference equations.

## 1 INTRODUCTION

In 2012, the Global Lung Function Initiative (GLI) published spirometric reference equations for worldwide use (Quanjer et al., 2012). These prediction equations were developed for four distinct ethnic groups: Caucasians (Whites); African‐Americans (Blacks); Northeast Asians; and Southeast Asians. The equations demonstrated that differences in Race/ethnicity existed and that race/ethnicity should be incorporated into prediction equations. For example, Black individuals have 12%–15% lower lung function than their White counterparts after controlling for age, sex, and height (Hankinson et al., 1999; Jonas et al., 2018; Kiefer et al., 2011; Lapp et al., 1974; Quanjer et al., 2012; Rossiter & Weill, 1974).

However, there has been efforts to remove race as a significant covariate in pulmonary function testing (Bhakta et al., 2022, 2023; Braun, 2021; Braun & Grisson, 2023; Regan et al., 2024; Vyas et al., 2020). Central to the argument is that race, which is a social construct, is an unreliable proxy for genetic differences (Ripp & Braun, 2017) and that self‐declaration of race may or may not corroborate with genetic diversity (Bryc et al., 2015; Lao et al., 2010). Reference equations using a race‐neutral approach have been developed (Bowerman et al., 2023a), and have been adopted and recommended by the American Thoracic Society (ATS) (Bhakta et al., 2023; Bowerman et al., 2023b).

Multiple societies recently published a research statement identifying research gaps around Race and pulmonary function interpretation (Marciniuk et al., 2023). Therefore, we retrospectively sought to examine discordance in obstructive and restrictive spirometry patterns between established race‐specific prediction equations to the 2023 GLI race‐neutral equation (Bowerman et al., 2023a). In addition, we assessed binary classification and model fit using our own developed race‐specific equations to equations without the race covariate.

## 2 METHODS

The study was approved by the Institutional Review Board at Loma Linda University (IRB#: 5210283) as a retrospective study. Informed consent was waived per 45 CFR 46.116(d). Spirometric data was extracted from patient visits between June 1, 2010, and August 31, 2021. Dedicated respiratory care staff performed testing with combined experience in pulmonary function testing of over 60 years, and ATS/ERS guidelines for all testing and quality control were followed (Miller, Crapo, et al., 2005; Miller, Hankinson, et al., 2005; Pellegrino et al., 2005). The dataset was screened and reviewed. Patients ≥18 years of age who self‐reported as White, Black, or American Hispanic at the time of testing were included in the dataset. Self‐identified Asians were excluded because there were fewer than 100 subjects. Patients with missing key data were excluded. In patients with a slow vital capacity (SVC) larger than the forced vital capacity (FVC), the slow vital capacity (SVC) was used to replace the FVC (Miller, Hankinson, et al., 2005). Only the first available PFT was used for patients with multiple visits.

### 2.1 Data analysis and reference equations

#### 2.1.1 Discordance

The following parameters were analyzed: Forced Expiratory Volume in 1 s (FEV_1_), FVC, FEV_1_/FVC ratio, obstruction (FEV_1_/FVC ≥ LLN and FVC ≥ LLN), and restrictive spirometric pattern (FEV_1_/FVC ≥ LLN and FVC < LLN). Discordance was determined as the percentage difference between abnormal parameters when comparing race‐specific to race‐neutral prediction equations. The 2012 GLI reference equation [1] was used as the race‐specific prediction equation for Black individuals, and the 1999 NHANES III equation [6] was used as race‐specific prediction equation for Hispanic individuals. Data was compared to 2023 GLI race‐neutral prediction equations. Values <LLN (<5th percentile or z‐scores < −1.645) were considered abnormal.

A Kappa statistical analysis was performed to examine the discordance in the LLN between equations. The discordance was calculated as 1 – *κ*, Where *κ* reflects concordance (McHugh, 2012). The categories of discordance are thus translated as: (1 – *κ*) < 0.1 = Negligible discordance; 0.1 ≤ (1 – *κ*) < 0.2 = Very low discordance; 0.21 ≤ (1 – *κ*) < 0.4 = low discordance; 0.41 ≤ (1 – *κ*) < 0.6 = moderate discordance; 0.61 ≤ (1 – *κ*) < 0.79 = high discordance; 0.8 ≤ (1 – *κ*) = very high discordance.

In the analysis of individuals with cardiopulmonary disease compared to those exhibiting average spirometry results, key metrics such as true positives, true negatives, false positives, and false negatives were determined using the GLI Race‐Specific equation as a benchmark (Wald & Bestwick, 2014). The Matthews Correlation Coefficient (MCC), a measure akin to Pearson's Correlation Coefficient but tailored for binary outcomes, was employed to assess these binary categories (Boughorbel et al., 2017; Chicco et al., 2021; Chicco & Jurman, 2020, 2023; Powers, 2011). This analysis produced four critical values: True Positive Rate (TPR), True Negative Rate (TNR), Positive Predictive Value (PPV), and Negative Predictive Value (NPV). These values collectively contribute to a comprehensive score representing the model's classification accuracy. The interpretation of the MCC is as follows: an MCC greater than 0.80 indicates a robust classification accuracy; an MCC between 0.60 and 0.80 suggests a moderately strong classification accuracy; an MCC between 0.30 and 0.50 is considered fair classification accuracy; and an MCC below 0.30 is deemed poor classification accuracy (Chan, 2003).

### 2.2 Development of best fit equations

The least absolute shrinkage and selection operator (LASSO) regression was implemented to identify the most important predictor variables for FEV_1_, FVC, and the FEV_1_/FVC ratio while minimizing prediction errors and overfitting. From the complete study dataset, only patients with a BMI between 18.5 and 34.9 kg/m^2^ and normal spirometry were used. Normal spirometry was defined as values ≥ LLN and ≤ULN (upper limit of normal) for FEV_1_/FVC ratio, FVC, and FEV_1_, based on 2023 GLI Race‐Neutral equations (Bowerman et al., 2023a).3771 subjects met the criteria: (2460 Whites, 376 Blacks, and 944 Hispanics, Figure S1, Table S1) while 132 patients (2%) were considered outliers. The covariates used in LASSO regression were age (18–95 years old), (Age)^2^, (Age)^3^, height (142–206 cm), (Height)^2^, weight (42–140 kg), sex, self‐identified race/ethnicity (either White, Black, or Hispanic).

Multiple linear regression was used to create best‐fit equations with normal spirometry values from the LASSO‐identified predictors. Models in which race/ethnicity was a significant covariate were compared to the same model but with that covariate removed using Bayesian Information Criterion (BIC). Models with a lower BIC fit better than those with higher BICs (Raftery, 1995). The difference in BIC was used to assess the necessity of race/ethnicity in the model. The interpretation of between‐model differences in BIC was taken from Raftery (1995) and is as follows: BIC difference of 0–2 = WEAK evidence or a 50%–75% probability that the model with the lower BIC is a better fit compared to the other models, BIC difference of 2–6 = POSITIVE evidence or 75%–95% probability that the model with the lower BIC is a better fit compared to other models, BIC difference of 6 to 10 = STRONG evidence or 95%–99% probability that the model with the lower BIC is a better fit compared to the other models, BIC difference is >10 = VERY STRONG evidence or >99% probability that the model with the lower BIC is a better fit compared to the other models. All independent predictors were evaluated via the variance inflation factor (VIF) to control for multicollinearity, with a VIF of 1 indicating a complete absence of multicollinearity, a VIF <5 indicating low collinearity, 5.0–9.9 indicating moderate collinearity, and >10 indicating high collinearity (Lüdecke et al., 2021). The 95% confidence interval (CI) for the VIF was produced for each predictor (Marcoulides & Raykov, 2019) and removed from the model when the upper bound of the VIF exceeded 10 (Johnston et al., 2018). A high VIF was ignored if it was determined to be due to a one quadratic effect and not a separate effect. Model assumptions were visually checked using the *performance* package in R (Lüdecke et al., 2021). This included a positive predictor check for systematic discrepancies between actual and simulated data and assessment of regression fit, linearity, homogeneity of variance, influential observations, multicollinearity, and normality of residuals. Any studentized residuals ≥3.00 were eliminated. In the models, the following rates were computed with Race/ethnicity either added or removed as the covariate: False Positive Rate (FPR) = False Positives (FP) ÷ [FP + True Negatives (TN)], False Negative Rate (FNR) = False Negatives (FN) ÷ [FN + True positive (TP)], False Discovery Rate (FDR) = FP ÷ [FP + TP], and False Omission Rate (FOR) = FN ÷ (FN + TN).

### 2.3 Prediction accuracy

To evaluate prediction accuracy, a repeated 10‐fold cross‐validation procedure was employed, with 1000 repetitions to minimize variability and provide a robust RMSE estimate. In comparing Blacks to Whites and Hispanics to Whites, the data was divided into 10 equal parts (folds). Each model (FEV_1_, FVC, FEV_1_/FVC ratio) was trained on 9 folds (90% of the data) and validated on the remaining fold (10%). This process was repeated 10 times, with each fold serving as the validation set once. The entire cross‐validation procedure was then repeated 1000 times, each time using a different random split of the data into 10 folds. Performance metrics, including RMSE and correlation, were averaged across all 1000 repeats, providing a robust estimate of the model's performance by accounting for variability in data splits. The reported correlation coefficient reflected the relationship between the actual and predicted values on the testing set. Additionally, the median, minimum, maximum, and 95% confidence intervals (CI) of the RMSE were provided, along with the median and 95% CI of the correlation coefficients between predicted and actual values.

### 2.4 Statistical software

SPSS IBM Statistics® Version 29 (IBM Corporation, Chicago, IL), Medcalc® Version 22.006 (Ostend, Belgium), the “R” language environment (version 4.3.0, April 21, 2023) (R Core Team, 2022), and Rstudio (2024.04.2, Build 764, June 5th 2024) were used for statistical analyses and for comparing outputs between software brands. A *p*‐value of <0.05 signified statistical significance.

## 3 RESULTS

In this set of 9147 patients, there were 5796 self‐identified Whites (54% females), 976 Blacks (64% females), and 2375 Hispanics (52% females) (Table 1). Fitted z‐scores for Table 1 are presented as additional material (Table S1).

**TABLE 1:** Subject characteristics of the full data used in the analysis of Table 2.

	Whites	Blacks	Hispanics
Males			
Number	2695	354	1142
Age (yrs)	61 (16) [18–95]	56 (16) [18–92]	53 (17) [18–91]
Weight (kg)	92.6 (21.6) [41.0.‐237.0]	94.0 (23.6) [45.0–231.0]	87.7 (22.2) [41.0–231.0]
Height (cm)	177 (8) [142–206]	178 (8) [157–203]	171 (8) [150–196]
BMI (kg/m^2^)	29.4 (6.4) [15.5–73.0]	29.6 (7.0) [16.3–79.9]	30.0 (6.7) [15.4–72.9]
FEV_1_ (L)	2.74 (1.01) [0.44–6.13]	2.47 (0.88) [0.50–4.83]	2.89 (0.94) [0.48–5.84]
FVC (L)	3.92 (1.15) [0.87–8.22]	3.44 (0.88) [1.32–6.55]	3.77 (1.09) [1.14–7.34]
FEV_1_/FVC	0.69 (0.13) [0.18–0.95]	0.71 (0.13) [0.23–0.94]	0.75 (0.10) [0.22–0.95]
Females			
Number	3101	622	1233
Age (yrs)	59 (17) [18–95]	56 (16) [18–92]	52 (18) [15–92]
Weight (kg)	79.0 (22.7) [40.0–246.0]	85.5 (24.5) [40.0–203.0]	77.7 (22.4) [41.0–202.0]
Height (cm)	163 (7) [142–189]	164 (7) [145–188]	158 (7) [142–180]
BMI (kg/m^2^)	29.7 (8.1) [15.4–90.4]	31.9 (8.6) [16.6–70.2]	31.0 (8.3) [15.4–80.9]
FEV_1_ (L)	2.06 (0.76) [0.34–4.49]	1.87 (0.62) [0.45–4.31]	2.12 (0.70) [0.41–4.39]
FVC (L)	2.80 (0.81) [0.80–5.83]	2.49 (0.70) [0.85–5.13]	2.67 (0.78) [0.80–5.33]
FEV_1_/FVC	0.72 (0.12) [0.21–0.95]	0.75 (0.11) [0.31–0.95]	0.79 (0.10) [0.21–0.95]

*Note*: Mean (SD) [minimum–maximum]. The full data set included 9147 subjects (normal and diseased).

### 3.1 Concordance and discordance

From the data presented in Table 1, the 1 – *κ* indicated a “very low discordance” for identifying airway obstruction between race‐specific and race‐neutral spirometry reference equations, as about 4%–5% of the data were discordant (see Table 2). The 1 – *κ* value also demonstrated a “low discordance” for identifying a restrictive spirometry pattern between race‐specific and race‐neutral spirometry reference equations, as about 5%–13% of the data were discordant (Table 2).

**TABLE 2:** The effectiveness of race‐specific versus race‐neutral spirometry reference equations.

GLI Whites vs. GLI race neutral (*n* = 5796)									
Spirometric parameters	Number (%) of discordant cases	1 – *κ*	MCC	F_1_ Score	PPV	NPV	FPR	FNR	FDR
Airway obstruction (17%)	252 (4%)	0.14 [0.12 to 0.16]	0.87	0.88	0.79	1.00	0.05	0.00	0.21
Restrictive spirometric pattern (17%)	291 (5%)	0.21 [0.18 to 0.23]	0.81	0.82	1.00	0.94	0.00	0.30	0.00
FEV_1_ < LLN (38% < LLN)	424 (7%)	0.16 [0.15 to 0.18]	0.85	0.89	1.00	0.90	0.00	0.19	0.00
FVC < LLN (25% < LLN)	413 (7%)	0.21 [0.19 to 0.22]	0.81	0.84	1.00	0.91	0.00	0.28	0.00

GLI Blacks vs. GLI race neutral (*n* = 976)									
	Number (%) of discordant cases	1 – *κ*	MCC	F_1_ Score	PPV	NPV	FPR	FNR	FDR
Airway obstruction (17%)	48 (5%)	0.19 [0.14 to 0.25]	0.82	0.83	0.98	0.95	0.00	0.27	0.02
Restrictive spirometric pattern (16%)	125 (13%)	0.36 [0.31 to 0.42]	0.68	0.71	0.55	1.00	0.15	0.00	0.45
FEV_1_ < LLN (37% < LLN)	120 (12%)	0.25 [0.21 to 0.29]	0.78	0.86	0.75	1.00	0.20	0.00	0.25
FVC < LLN (23% < LLN)	162 (17%)	0.37 [0.32 to 0.42]	0.68	0.74	0.59	1.00	0.22	0.00	0.41

NHANES Hispanics vs. GLI race neutral (*n* = 2375)									
	Number (%) of discordant cases	1 – *κ*	MCC	F_1_ Score	PPV	NPV	FPR	FNR	FDR
Airway obstruction (12%)	87 (4%)	0.18 [0.15 to 0.22]	0.82	0.84	0.89	0.97	0.01	0.21	0.11
Restrictive spirometric pattern (20%)	206 (9%)	0.29 [0.25 to 0.33]	0.72	0.76	0.86	0.92	0.03	0.32	0.14
FEV_1_ < LLN (37% < LLN)	285 (12%)	0.27 [0.24 to 0.30]	0.75	0.81	0.99	0.84	0.00	0.32	0.01
FVC < LLN (26% < LLN)	230 (10%)	0.27 [0.24 to 0.30]	0.74	0.79	0.91	0.90	0.03	0.30	0.09

GLI other or mixed vs. GLI race neutral (*n* = 9148)									
	Number (%) of discordant cases	1 – *κ*	MCC	F_1_ Score	PPV	NPV	FPR	FNR	FDR
Airway obstruction (19%)	260 (3%)	0.10 [0.08 to 0.11]	0.91	0.92	0.97	0.97	0.01	0.12	0.03
Restrictive spirometric pattern (15%)	270 (3%)	0.12 [0.10 to 0.13]	0.88	0.90	0.90	0.98	0.02	0.10	0.10
FEV_1_ < LLN (31% < LLN)	236 (3%)	0.06 [0.05 to 0.07]	0.94	0.96	0.96	0.98	0.02	0.04	0.04
FVC < LLN (22% < LLN)	298 (3%)	0.10 [0.08 to 0.11]	0.90	0.92	0.95	0.97	0.01	0.09	0.05

*Note*: (1 – *κ*) < 0.1 = Negligible discordance; 0.1 ≤ (1 – *κ*) < 0.2 = Very low discordance; 0.21 ≤ (1 – *κ*) < 0.4 = Low discordance; 0.41 ≤ (1 – *κ*) < 0.6 = Moderate discordance; 0.61 ≤ (1 – *κ*) < 0.79 = High discordance; 0.8 ≤ (1 – *κ*) = Very high discordance. The percentage of subjects below the LLN is presented in the first column and pertains to the Race‐Specific percentage that is < LLN.

Abbreviations: F_1_ Score = Harmonic mean of precision and sensitivity; FDR, False Discovery Rate; FNR, False Negative Rate or the Miss Rate; FPR, False Positive Rate or the Probability of a False Alarm; MCC, Matthews Correlation Coefficient; NPV, Negative Predictive Value or Specificity; PPV, Positive Predictive Value or Precision.

### 3.2 Development of best fit spirometry equations from LLU data

Subjects with a BMI from 18.5 to 34.9 kg/m^2^ and normal spirometry were selected to create best‐fit spirometry reference equations. After screening for normal spirometry values and removing outliers (2.4%, or 132 patients), the dataset was reduced from 9147 to 3771 subjects (2460 Whites, 367 Blacks, and 944 Hispanics, Table 3, Figure S1).

**TABLE 3:** Subjects used in the development of the reference equations to examine Race/ethnic influences in spirometry.

	Whites	Blacks	Hispanics
Males			
Number	1108	136	457
Age (yrs)	60 (18) [18–95]	55 (17) [18–92]	52 (17) [18–90]
Weight (kg)	87.3 (14.2) [43.0.‐130.0]	89.4 (13.3) [52.0–140.0]	81.5 (13.4) [48.0–122.0]
Height (cm)	178 (8) [149–206]	178 (8) [157–203]	170 (8) [150–193]
BMI (kg/m^2^)	27.7 (3.8) [18.5–34.9]	28.3 (3.7) [19.1–34.8]	28.1 (3.7) [18.7–34.9]
FEV_1_ (L)	3.32 (0.80) [1.59–6.13]	3.00 (0.65) [1.62–4.52]	3.35 (0.75) [1.41–5.47]
FVC (L)	4.41 (0.93) [2.21–7.74]	3.86 (0.73) [2.17–5.38]	4.25 (0.86) [1.85–6.45]
FEV_1_/FVC	0.75 (0.06) [0.59–0.93]	0.78 (0.06) [0.65–0.92]	0.79 (0.06) [0.63–0.95]
Females			
Number	1352	231	487
Age (yrs)	58 (18) [18–95]	56 (17) [18–90]	50 (18) [18–92]
Weight (kg)	71.6 (13.0) [42.0–121.0]	74.0 (12.5) [47.0–108.0]	68.7 (11.9) [42.0–109.0]
Height (cm)	163 (7) [142–189]	163 (6) [145–188]	158 (7) [142–180]
BMI (kg/m^2^)	26.8 (4.2) [18.6–34.9]	27.7 (4.2) [18.6–34.9]	27.5 (4.1) [18.6–34.9]
FEV_1_ (L)	2.43 (0.63) [0.98–4.49]	2.16 (0.49) [1.11–2.48]	2.41 (0.59) [0.90–4.36]
FVC (L)	3.11 (0.71) [1.38–5.39]	2.71 (0.53) [1.40–4.51]	2.94 (0.66) [1.17–5.33]
FEV_1_/FVC	0.78 (0.06) [0.61–0.95]	0.80 (0.06) [0.62–0.95]	0.82 (0.06) [0.65–0.94]

*Note*: Mean (SD) [minimum‐maximum]. The equations were developed from subjects with normal spirometry from the full data (*n* = 9147) to come up with *n* = 3771 identified in this table.

Six best‐fit models using LASSO‐identified predictors of FEV_1_, FVC, and FEV_1_/FVC are presented in Tables S2–S4. Race accounted for ≤4% of the total shared variance. However, removing Race from any model increased the BIC by at least 10 units (Table S5). This demonstrates a greater than 99% probability that all the models that includes Race as a covariate was a better fit compared to models with Race removed as a covariate.

Prediction accuracy was assessed via the root‐mean‐square‐error (RMSE). The RMSE was lower (0.33 vs. 0.36) when race‐specific models for Blacks (compared to Whites) were used for FEV_1_ and FVC (Table S5) when compared to race‐neutral equation (*p* < 0.001). High correlation coefficients were found between predicted and test data, repeated 1000 times, validating the models (Table S6). The assumptions of linearity, homogeneity of variance, influential observations, multicollinearity, and normality of residuals are presented in Figures S2–S7.

When other covariates were controlled, the difference in predicted FEV_1_ between Whites and Blacks increased as height increased, at about +0.27 L at 140 cm tall, to about +0.48 L at 185 cm tall, favoring Whites (Table S2). However, the difference in predicted FEV_1_ between Whites and Hispanics remained stable throughout all heights and ages, displaying a predicted higher FEV_1_ in Whites compared to Hispanics by about +0.56 L (Table S3).

When age and sex were controlled, the difference in predicted FVC in Whites compared to Blacks increased as height increased, from about +0.40 L at 140 cm to about +0.70 L at 185 cm tall, favoring Whites. When controlling for age and sex, FVC was +0.25 L more in Whites compared to Hispanics at a height of ~140 cm. However, this racial difference in predicted FVC between Whites and Hispanics is eliminated when height exceeds 175 cm.

**FIGURE 1:**
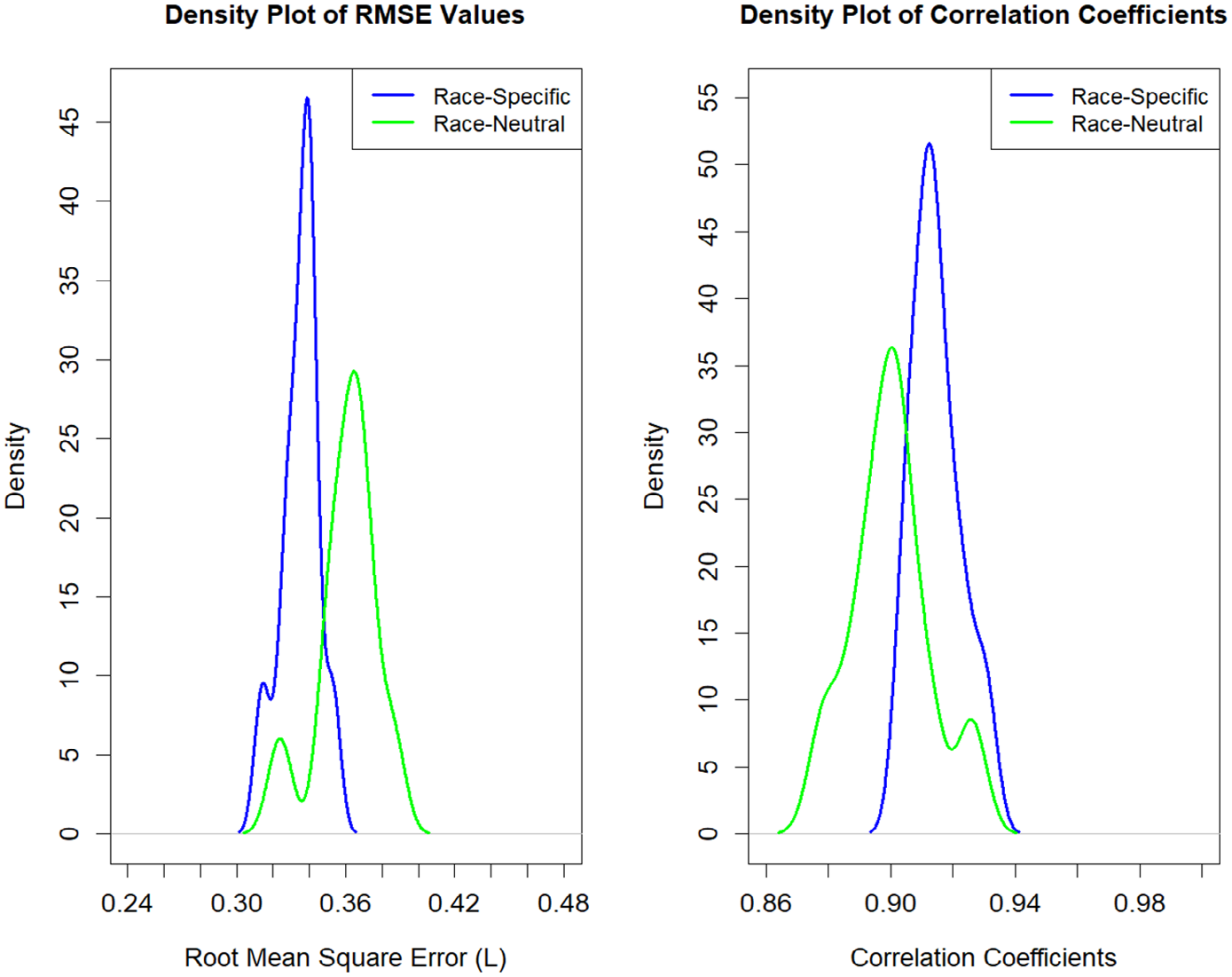
Density plot of root mean square error (RMSE) values between race‐specific and race‐neutral equations for FEV_1_, (Blacks vs. Whites). Race‐specific equation (Blue Line): This equation includes race as a covariate, with race coded as 0 for Mexican and 1 for White. The race‐neutral equation (Green Line) does not include race as a covariate and treats all subjects as one race. Left panel: The density plot shows that the race‐specific reference equation tends to have lower RMSE values compared to the race‐neutral reference equation, as indicated by the blue line being shifted to the left of the green line. The peak density for the race‐specific equation is higher and more concentrated around lower RMSE values, suggesting better model performance compared to the race‐neutral reference equation. The spread of the RMSE values is narrower for the race‐specific equation, indicating more consistent performance. Permutation test results: RMSE Difference = 0.025 L, two‐sided *p*‐value = 0.004. Thus, this difference is unlikely to have occurred by chance. However, the bootstrapped test results did not show a statistically significant difference between the equations (two‐sided, *p* = 0.567), implying that this small difference may not be practically meaningful. Thus, in real‐world applications, the choice between race‐specific and race‐neutral equations may not be so important for FEV_1_ when comparing Blacks versus Whites. Right panel: The blue line (race‐specific equation) is shifted to the right compared to the green line (race‐neutral equation), indicating that the race‐specific equation generally has higher correlation coefficients (permutation test results, correlation difference = −0.013, two‐sided *p*‐value = 0.008). This implies that the correlations between actual values and the predicted values on the testing set were closer using the race‐specific equation than the race‐neutral equation. However, the bootstrap results did not show a statistically significant difference between the two equations (*p* = 0.598), also implying that this small difference may not be practically meaningful. On the other hand, airway obstruction is defined by a combination of FEV_1_/FVC and FVC.

**FIGURE 2:**
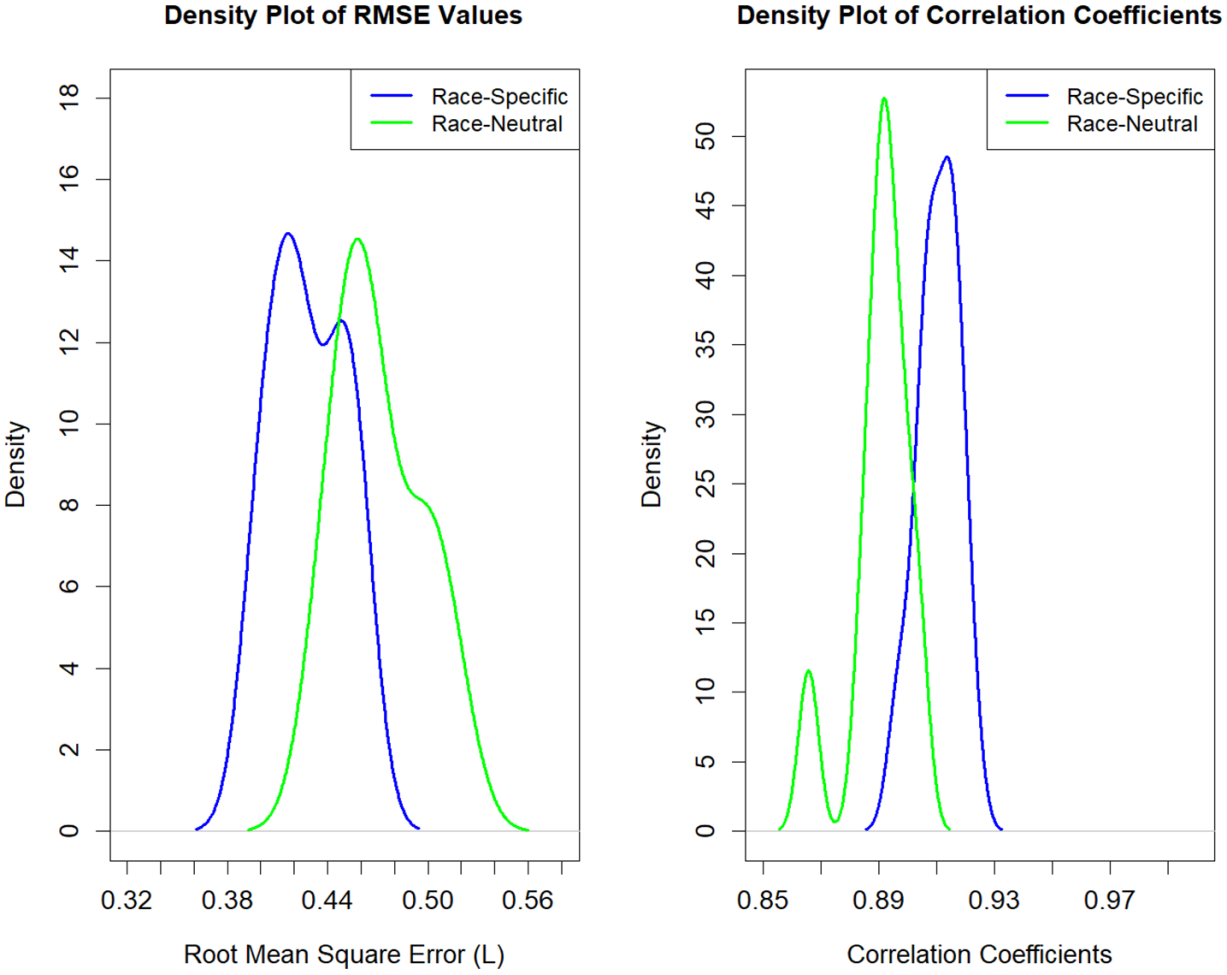
Density plot of root mean square error (RMSE) values between race‐specific and race‐neutral equations for FVC (Blacks vs. Whites). Race‐specific equation (Blue Curve): This equation includes race as a covariate, with race coded as 0 for Mexican and 1 for White. The race‐neutral model (Green Curve) does not include race as a covariate and treats all subjects as one race. Left panel: The density plot shows that the race‐specific equation tends to have lower RMSE values compared to the race‐neutral equation, as indicated by the blue line being shifted to the left of the green line. The peak density for the race‐specific equation is and more concentrated around lower RMSE values, whereas the density plots for the race‐neutral equation is wider. This suggests that the race‐specific equation has a better and more consistent model performance compared to the race‐neutral equation. Permutation test results: RMSE Difference = 0.038 L, two‐sided *p*‐value = 0.0011. Thus, the difference between equations is unlikely to have occurred by chance. However, the bootstrapped test results did not show a statistically significant difference between the equations (two‐sided, *p* = 0.527), implying that this small difference may not be practically meaningful. Thus, in real‐world applications, the choice between race‐specific and race‐neutral equations for FVC may not be so important when comparing Blacks versus Whites. Right panel: The blue line (race‐specific equation) is shifted to the right compared to the green line (race‐neutral equation), indicating that the race‐specific equation generally has higher correlation coefficients (permutation test results, correlation difference = −0.019, two‐sided *p*‐value = 0.0008). This implies that the correlations between actual values and the predicted values on the testing set were closer using race‐specific equations. However, the bootstrap results did not show a statistically significant difference between the two equations (*p* = 0.490), implying that this small difference may not be practically meaningful.

After validating the best‐fit spirometry reference equations (Tables S5 and S6) from the data in Table 3, we compared them to equations without race as a covariate. The RMSE derived from permutation testing was significantly lower for both FEV_1_ (*p* = 0.0004) and FVC (*p* = 0.0011) when race‐specific equations for Blacks and Whites were used compared to Race‐Neutral equations (Figures 1 and 2, Table S5). However, the bootstrapped test results did not reveal a statistically significant difference in RMSE between the race‐specific (Black vs. White) and race‐neutral models for FEV_1_ and FVC.

As for the RMSE and correlation coefficients for FEV_1_/FVC ratio, both permutation and bootstrapped results were not statistically different between any race‐specific and any race‐neutral models (Figures S8 and S11, Tables S5 and S6). There was also no statistical difference in RMSE or correlation coefficients when comparing race‐specific (Hispanics vs. Whites) and race‐neutral models for FEV_1_ and FVC (Figures S9 and S10, Tables S5 and S6).

When race was eliminated from Models 1 to 6 (Table S5), there was a “moderate” and “high” discordance for identifying a restrictive spirometry pattern in Hispanics and Blacks, respectively (Table 4). Furthermore, the FNR for identifying airway obstruction in Blacks and Hispanics was ~44% when race was omitted as a covariate from prediction equations (Table 4). As well, the FNR was 43%–44% for identification of airway obstruction in Blacks and 63% for identifying a restrictive spirometry pattern in Whites Hispanics when race was omitted as a covariate (Table 4). Moderate to high discordance was observed for identifying FEV_1_ and FVC below the LLN in Blacks (Table 5).

**TABLE 4:** The differences in obstructive and restrictive classifications between developed reference equations with and without race as a covariate (Model 2 and 5, Blacks; Model 4 and 6, Hispanics).

Using best fit equations from Model 2 and 5	Obstruction in Blacks (*n* = 367) (4%)	Obstruction in Whites (*n* = 2460) (5%)	Restrictive spirometry pattern in Blacks (*n* = 367) (3%)	Restrictive spirometry pattern in Whites (*n* = 2460) (4%)
False Positive Rate (False Alarm)	0%	1%	26%	0%
False Discovery Rate (Percentage of those below the LLN that are incorrect)	0%	9%	90%	0%
False Negative Rate (Miss Rate)	44%	0%	0%	63%
False Omission Rate (Percentage of those labeled not restrictive or non‐obstructive that are incorrect)	2%	0%	0%	2%
AUC	0.78	0.99	0.87	0.68
MCC	0.74	0.95	0.27	0.60
Discordance	Low discordance	Negligible discordance	High discordance	Low discordance
Number (%) of the sample affected when Race is omitted from the prediction model	7 (1.9) %	12 (0.5) %	137 (37.3) %	49 (2.0) %

Using best fit equations from Model 4 and 6	Obstruction in Hispanics (*n* = 944) (5%)	Obstruction in Whites (*n* = 2460) (5% < LLN)	Restrictive spirometry pattern in Hispanics (*n* = 944) (1.5%)	Restrictive spirometry pattern in Whites (*n* = 2460) (5%)
False Positive Rate (False Alarm)	0%	0%	1%	1%
False Discovery Rate (Percentage of those below the LLN that are incorrect)	0%	3%	47%	17%
False Negative Rate (Miss Rate)	43%	3%	36%	3%
False Omission Rate (Percentage of those ≥ LLN that are incorrect)	2%	0%	1%	0%
AUC	0.79	0.98	0.82	0.98
MCC	0.75	0.97	0.58	0.89
Discordance	Low discordance	Negligible discordance	Moderate discordance	Very low discordance
Number (%) of the sample affected when Race is omitted from the prediction model	21 (2.2%)	8 (0.3) %	13 (1.4) %	32 (1.3) %

*Note*: The LLN is <5th percentile, or a z‐score more negative than −1.645.

Abbreviations: AUC, area under the ROC curve; FDR, false discovery rate; FNR, false negative rate (type II error rate); FOR, False omission rate, or the probability that the value is <LLN, provided that the test result is ≥LLN; FPR, false positive rate (Type I error rate); MCC, Matthews Correlation Coefficient.

**TABLE 5:** The differences in the classifications of FEV_1_ and FVC being < LLN when Race is included or excluded from the developed reference equations (Models 1–4).

Using best‐fit equations from Models 1 and 2	FEV_1_ Blacks (*n* = 367) (2% < LLN)	FEV_1_ Whites (*n* = 2460) (3% < LLN)	FVC Blacks (*n* = 367) (3% < LLN)	FVC Whites (*n* = 2460) (4% < LLN)
False Positive Rate (False Alarm)	23%	0%	26%	4%
False Discovery Rate (Percentage of those below the LLN that are incorrect)	92%	0%	90%	74%
False Negative Rate (Miss Rate)	0%	74%	0%	63%
False Omission Rate (Percentage of those ≥LLN that are incorrect)	0%	2%	0%	2%
AUC	0.89	0.63	0.87	0.66
MCC	0.25	0.50	0.27	0.28
Discordance	High discordance	Moderate discordance	High discordance	High discordance
Percent of the sample affected when Race is omitted from the prediction model	22.6%	2.4%	25.6%	2.3%

Using best‐fit equations from Models 3 and 4	FEV_1_ Hispanics (*n* = 944) (2% < LLN)	FEV_1_ Whites (*n* = 2460) (4% < LLN)	FVC Hispanics (*n* = 944) (2% < LLN)	FVC Whites (*n* = 2460) (5% < LLN)
False Positive Rate (False Alarm)	0%	0%	1%	0%
False Discovery Rate (Percentage of those below the LLN that are incorrect)	15%	5%	47%	3%
False Negative Rate (Miss Rate)	27%	1%	36%	8%
False Omission Rate (Percentage of those ≥ LLN that are incorrect)	0%	0%	1%	0%
AUC	0.87	0.99	0.82	0.96
MCC	0.79	0.97	0.58	0.94
Discordance	Low Discordance	Negligible Discordance	Moderate Discordance	Negligible Discordance
Percent of the sample affected when Race is omitted from the prediction model	0.6%	0.2%	1.4%	0.5%

*Note*: The LLN is considered as a z‐score of ‐1.645, so any z‐score more negative than ‐1.645 is below the LLN.

Abbreviations: AUC, area under the ROC curve; FDR, false discovery rate; FNR, false negative rate (type II error rate); FOR, False omission rate, or the probability that the value is <LLN, provided that the test result is ≥LLN; FPR, false positive rate (Type I error rate); MCC, Matthews Correlation Coefficient.

## 4 DISCUSSION

In recent years, use of race correction in pulmonary function testing interpretation and in clinical algorithms (Bhakta et al., 2022, 2023; Braun, 2021; Braun & Grisson, 2023; Regan et al., 2024; Vyas et al., 2020) has been questioned and addressing it has become a priority of the U.S. Government (Khazanchi et al., 2022). We therefore decided to examine discordance, binary classification, and model fit between race‐predicted and race‐neutral spirometry prediction equations from a large dataset at LLUMC. In the first approach, we examined the measured spirometric values obtained from ~9000 patients, and retrospectively applied race‐predicted spirometric reference equations [1, 6] to identify those that were below the LLN for obstructive, restrictive, and mixed respiratory patterns. Then we examined the discordance of those below the LLN when the GLI race neutral equations were used (Bowerman et al., 2023a). We found that there was a 13% discordance in identifying a restrictive spirometric pattern between GLI race‐specific equations compared to GLI race‐neutral equations in the Black population. On a positive note, there was only a 1%–3% discordance for identifying obstruction between race‐specific and race‐neutral prediction equations.

In the second approach, we developed and rigorously validated our own prediction equations using patients with normal spirometry from the same dataset. The Bayesian Information Criterion (BIC) was consistently lowest for all models (1–6) when race was included as a covariate, and it increased significantly when race was excluded. Specifically, models that included race had a BIC more than 10 units lower than those without it (Table S5), indicating a better fit. The BIC penalizes models for having more parameters, so the fact that all models that includes Race has a lower BIC despite having more parameters compared to race‐neutral models suggests that the improvement in fit due to including Race outweighs the penalty for the additional complexity. In other words, Race is an important factor in predicting the outcome, and the model is better with it included, even after accounting for the increased model complexity.

Although race/ethnicity accounted for ≤4% of the total R^2^ in our models, removing race (Black vs. White) resulted in an increase in the root mean square error (RMSE) for both FEV_1_ and FVC (Table S5). We observed that FEV_1_ and FVC were higher in Whites compared to Blacks by 0.27 and 0.39 L, respectively, at a height of 140 cm, and by 0.48 and 0.68 L, respectively, at a height of 185 cm. These differences align with the findings of Burney and Hooper (2012), who reported similar differences even after adjusting for several confounders (Burney & Hooper, 2012).

When the RMSE was bootstrapped, no statistically significant difference was found between race‐specific (Black vs. White) and race‐neutral equations for FEV_1_ and FVC, suggesting that the observed RMSE difference may not be practically significant. This could imply that the choice between race‐specific and race‐neutral models might not be critical for FEV_1_ or FVC in real‐world applications. However, we approach this conclusion with caution, as the false‐negative rate (FNR) for diagnosing airway obstruction in Blacks is 44% when using a race‐neutral approach (Table 4). Additionally, despite the non‐significant RMSE differences from bootstrapping, race‐neutral equations can increase the prevalence of restriction in Blacks. Since restriction can be defined by a combination of the FEV_1_/FVC ratio and FVC (Moffett et al., 2023), even slight differences in RMSE between race‐specific and race‐neutral models can influence the lower limit of normal (LLN) in each equation, potentially leading to incorrect conclusions in diagnostic algorithms (i.e., like in the interpretation standards (Stanojevic et al., 2022)) and compounding errors along the algorithm.

Other data has shown that by the prevalence of restriction can increase by approximately 11% when race‐neutral models are used (Moffett et al., 2023). Our full dataset that included diseased patients (Table 1) shows a similar ~13% absolute increase in the prevalence of restriction [100·((278–153)/976)] when GLI Global (race‐neutral) equations were used instead of GLI race‐specific equations for Blacks. However, the interpretation of what the increase means differs between studies. We interpreted the increase in the prevalence of restriction as an *increase* in the number of FPs (false alarms) by 15% when race‐neutral reference equations were used in Blacks (Table 2). Moffett and colleagues, however, indicate that race‐neutral equations *reduce* the number of false negatives for identifying restriction (Moffett et al., 2023). Yet, as BIC increases (i.e., model fit is worse) in all prediction models when race is removed as a covariate in our results (Table S5), adding a race covariate improves all models' fit to the data at hand. Thus, it can be argued that based on our results that using a race‐neutral approach seems to promote health disparities by increasing the FPR for identifying lung restriction in Blacks. Therefore, further investigation is warranted in this area.

In the same vein, work by other investigators show that the prevalence of restriction decreased by ~5% in White individuals when race‐neutral equations were used (Moffett et al., 2023). We also demonstrated this same decrease leading to a false negative rate of 30% (Table 2). Again, these findings imply that using race‐specific equations in Whites increases the number of false positives compared to race‐neutral equations (Moffett et al., 2023). However, BIC increased (i.e., model fit is worse) in all prediction models when race is removed as a covariate as seen in our data (Table S5), which would imply that the FNR of 30% would be correct when using a race‐neutral approach.

Furthermore, Ekstrom and Mannino (2022) analyzed NHANES spirometry data (collected 2007–2012) (Ekstrom & Mannino, 2022). Using GLI race‐specific equations on Blacks, FEV_1_ below LLN was 37%; with Black‐specific equations, ~9%. This is due to Whites having larger FEV_1_ (~400 mL), which increases with height (Table S2). Ekström & Mannino interpret Black equations identifying a quarter of cases as underdiagnosis; it can also be interpreted as ~30% overdiagnosis using White equations for Blacks, as removing race/ethnicity from the FEV_1_ covariate raises BIC (Table S5).

The potential clinical impact of using race‐neutral equations was evaluated by Bonner et al. (2023) in a recent quality improvement study (Bonner et al., 2023). Surgeons were randomized to the race‐neutral versus race‐corrected PFT interpretation. Results demonstrated that surgeons were less likely to recommend potentially curative lobectomy for a lung nodule to African Americans when using race‐neutral PFT interpretation due to the disparate predicted post‐operative spirometric values yielded by the two equations (Bonner et al., 2023). To contextualize the difference at a population level, there are 35,665,417 African Americans in the United States ≥18 years of age.^1^ Given that the number of lung function tests is likely 34.7/10,000 (Li et al., 2019), about 123,759 lung function tests in Blacks occur annually in the United States. If race‐neutral spirometry equations are used, then, at best, ~18,564 incorrect diagnoses of restriction will occur annually (i.e., FPR = 0.15 or 15% in Table 2). Similarly, as the FPR for airway obstruction diagnosis is about 5% in Whites (Table 1), then, at best, ~34,500 incorrect diagnosis of obstruction will occur annually in the United States.^2^ In the Hispanic population, using the GLI race‐neutral equation resulted in a 21% FNR compared NHANES race‐Specific Equation (Table 2). Thus, In the United States, ~36,000 Hispanics could be falsely labeled as having no airway obstruction.^3^

Other researchers have supported the use of race‐specific equations. In a recent study (Moitra et al., 2024), the GLI Global (race‐neutral) spirometry reference equations [16] were compared with equations derived from over 1000 healthy Indian adults (≥18 years of age) (Chhabra et al., 2014). Moitra and colleagues found that the median FEV_1_, FVC, and FEV_1_/FVC z‐scores were 1.02, 0.74, and 0.27 units lower, respectively, when using the GLI Global equations compared to race‐specific equations for the Indian population. Similarly, Forno and colleagues observed that the mean FEV_1_ and FVC z‐scores in Black children were 0.81 and 0.91 units lower, respectively, when using the GLI Global instead of GLI race‐specific equations (Forno et al., 2024). They also noted that adopting the GLI Global reference equations could likely alter treatment in Black children with lung diseases (Forno et al., 2024). Although Brems and colleagues advocate for the use of race‐neutral reference equations, they also reported more negative FEV_1_ z‐scores in Blacks and more positive FEV_1_ z‐scores in Whites when using the GLI Global reference equations compared to GLI race‐specific equations (Brems et al., 2024). Our findings align with these observations, as we also demonstrate lower fitted FEV_1_ and FVC z‐scores in Blacks and more positive FEV_1_ z‐scores in Whites when using the GLI Global equations instead of GLI race‐specific equations (Table S1).

Furthermore, pulmonary diffusing capacity interpretation can also be affected using race‐neutral instead of race‐specific equations. Gochicoa‐Rangel et al. (2024) has advocated the use of race‐specific models in her country of Mexico. She and her colleagues revealed that using race‐specific reference equations resulted in superior accuracy compared to race‐neutral models (Gochicoa‐Rangel et al., 2024). For example, there she showed a 3%–6% FPR in Mexican Hispanics and a 20%–49% FNR in white subjects when race‐neutral reference equations were used instead of the race‐specific equations (Gochicoa‐Rangel et al., 2024).

While fitted z‐scores are usually different when using GLI Global versus GLI race‐specific equations, predicting mortality from any cause are similar between the two equations. In nearly 370,000 participants, it was shown that the area under the Kaplan Meier curve for 10‐year any‐cause mortality vs. fitted FEV_1_ z‐scores were similar in Whites, Blacks, and Hispanics, regardless of which equation was used (Diao et al., 2024). Despite the enormous online supplement provided in that study (Diao et al., 2024), there was no assessment of FVC or FEV_1_/FVC z‐scores and its association to mortality. Indeed, when they used GLI Global reference equations, the false positives increased in the Black population (Diao et al., 2024), although it is unclear for which spirometric variable had the increase in false positives. In our study, the FPR was 15% when identifying a restrictive spirometric pattern compared to GLI race‐specific equations (Table 2).

Due to inaccuracies, inconsistencies, and societal and healthcare implications of race‐specific prediction equations, the ATS in 2023, has recommended everyday use of race‐neutral prediction equations (Bhakta et al., 2023; Bowerman et al., 2023b), the long‐term impact of which is at best, uncertain. In a study by Harber et al. (1983), adjustment for race and sex had significant effects in the overall number of persons declared “disabled”. Although our study did not evaluate clinical events, the mathematical analyses found race to be a significant independent variable; removal of which from the predictive models resulted in increase in discordance and a poorer model fit. Instances may exist where the harm caused by use of race‐specific equations does not outweigh the benefits of using it. One such instance may be in relation to height. In our data, the mathematical influence of Race on discordance increases with the height of subjects. If this mathematical influence is corroborated by clinical outcomes in larger studies, there may be a certain height that could trigger the use of race‐specific versus race‐neutral equations. Further studies are warranted as well in this area.

The study has several limitations. First, the fact that the data collection was from one geographical area collected at a single center. Second, we did not have enough data to assess other races/ethnicities, and questions remain as to the significance and accuracy of self‐reported race. Third, we did not evaluate TLC of the complete dataset of 9147 patients to confirm restriction (as no GLI equations exist for TLC in Blacks and Hispanics). Therefore, since our study included a model that we created, we strongly recommend future studies to evaluate our proposed model and in different geographical locations to confirm our proposed models, results, and findings.

In conclusion, this study shows that while all the spirometric reference equations that include race fits the training data better than race‐neutral equations (as reflected by the lower BIC in race‐specific models), in some cases, the additional complexity introduced by including race does not necessarily translate to better out‐of‐sample predictions. In other words, the race covariate improves models' fit to the data at hand, but this improvement may not significantly enhance a model's ability to generalize to new data, as both equations perform similarly in terms of RMSE. Thus, the takeaway is that while all spirometric reference equations that includes race is statistically preferred based on BIC, its practical predictive power may not be much different from simple models, according to cross‐validation results. Nonetheless, restriction and obstruction is defined by a combination of the FEV_1_/FVC ratio and FVC (Stanojevic et al., 2022), so even slight differences in RMSE between race‐specific and race‐neutral models can influence the lower limit of normal (LLN) in each equation, potentially leading to incorrect conclusions in diagnostic algorithms (i.e., like in the interpretation standards (Stanojevic et al., 2022)) and compounding errors along the algorithm. We find that there is discordance in the interpretation of obstructive and restrictive patterns between race‐specific and GLI Global (race‐neutral) equations. Also, we found that when race is removed as a covariate in our prediction models, the FDR and FNR are high, and misclassification rate increases. This discordance in race‐specific and race‐neutral equations may have significant clinical repercussions. Thus, further studies are warranted to evaluate and confirm our findings.

## AUTHOR CONTRIBUTIONS

Conceptualization: GSZ, AA, PCG. Methodology: GSZ. Software: GSZ. Validation: GSZ. Formal analysis: GSZ. Investigation: AA, PCG, MHT, GSZ, JDA, SE, SBT. Resources: MHT, AA, PCG. Data curation: MHT, GSZ. Writing – original draft: GSZ. Writing – review and editing: GSZ, SE, AA, SBT, MHT, JDA, PCG. Visualization: GSZ. Supervision: AA, PCG. Project administration: AA, MHT.

## FUNDING INFORMATION

This research did not receive any specific grant from funding agencies in the public, commercial, or not‐for‐profit sectors.

## CONFLICT OF INTEREST STATEMENT

All authors report no conflicts of interest.

## ETHICS STATEMENT

The study was approved by the Institutional Review Board at Loma Linda University (IRB#: 5210283) as a retrospective study.

## Supporting information


Appendix S1.

## Data Availability

The data that support the findings of this study are available from the corresponding author upon reasonable request.

## Footnotes

^1^

https://www.census.gov/quickfacts/fact/table/US. 35,664,417 Blacks >18 years old in U.S. (July 2023).

^2^

https://www.census.gov/quickfacts/fact/table/US. 197,989,964 Whites >18 years old in U.S. (July 2023). About 687,025 PFTs per year, ×0.05 = 34,351 false positive airway obstruction classifications per year.

^3^

https://www.census.gov/quickfacts/fact/table/US. 50,087,527 Hispanics >18 years old in U.S. (July 2023). About 173,804 PFTs per year, ×0.21 = 36,182 false negative airway obstruction classifications per year.
