# Supplementary material for: Examining discordance in spirometry reference equations: A retrospective study
Source: Physiol Rep. 2025 Feb 26;13(5):e70212. doi: 10.14814/phy2.70212 (PMC11865334; doi:10.14814/phy2.70212)

**Online Supplement**

**Examining Discordance in Spirometry Reference Equations: A Retrospective Study**

^1,2^Gerald S. Zavorsky, ^2^Sherif Elkinany, ^2,4^Abdullah Alismail; ^4^Suman B. Thapamagar, ^4,5^Michael H. Terry, ^3,4^James D. Anholm, ^2,6^ Paresh C. Giri

^1^ Department of Physiology and Membrane Biology, University of California, Davis, Davis, CA

^2^ Department of Cardiopulmonary Sciences, Loma Linda University, Loma Linda, CA

^3^ Division of Pulmonary, Critical Care, Hyperbaric, and Sleep Medicine, Lima Linda University Veterans Administration, Loma Linda, CA

^4^Department of Medicine, School of Medicine, Loma Linda University, Loma Linda, CA,

^5^ Department of Respiratory Care, Loma Linda University Medical Center, Loma Linda, CA

^6^Beaver Medical Group (Optum), Redlands, CA

Gerald Stanley Zavorsky^1,2^ ^†^ ORCID: **0000-0002-4473-1601.**

**Abdullah Alismail^2,4^ ORCID: 000-0002-7844-8943.**

**Suman Thapamager^4^ ORCID: 0000-0003-4158-8085.**

**Michael H. Terry^4,5^ ORCID: 0000-0002-1561-2443**

James D. Anholm^3,4^ ORCID: **0000-0002-5699-3481.**

**Paresh C. Giri ORCID^6^: 0000-0002-0271-8572.**

**This supplement has been peer-reviewed.**

Address for Correspondence:

**Corresponding author:**

Abdullah Alismail, PhD, RRT, FCCP, FAARC

Associate Professor

Department of Cardiopulmonary Sciences, Department of Medicine

Loma Linda University Health, [aalismail@llu.edu](mailto:aalismail@llu.edu)

Statistical related questions can be directed to: [gszavorsky@ucdavis.edu](mailto:gszavorsky@ucdavis.edu)

**Information about data extraction.**

The data was extracted from the ULTIMA PFX^®^ pulmonary function system (Medical Graphics Corporation, Saint Paul, MN) using BreezeSuite™ cardiorespiratory diagnostic software version 7.1 (Service Pack 2) upgraded through version 8.4 (Service Pack 3).

**R packages used in this study.**

R packages *lmtest* (version 0.9-40) *performance* (version 0.12.4), *patchwork* (version 1.2.0), *AICcmodeavg* (version 2.3-3), *randomForest* (version 4.7-1.1), *relaimpo* (version 2.2-7) and *glmnet* (version 4.1-8).

**Table S1.** Z-scores based on the data from Table 1 of the main article.

|  | **Blacks (n=976)** | |  |
| --- | --- | --- | --- |
| **Variable** | **GLI-Race Specific reference equations** | **GLI Global reference equations** | **Difference**  **(GLI Global minus GLI Race Specific)** |
| FEV_1_ z-scores | –1.21 (1.44) | –1.65 (1.22) | –0.55 (0.20)  [–0.56, –0.53] |
| FVC z-scores | –0.82 (1.31) | –1.37 (1.18) | –0.45 (0.25)  [–0.47, –0.43] |
| FEV_1_/FVC z-scores | –0.84 (1.40) | –0.80 (1.33) | +0.03 (0.13)  [0.03, 0.04] |
|  | **Whites (n=5796)** | |  |
|  | **GLI-Race Specific reference equations** | **GLI-Global reference equations** | **Difference**  **(GLI Global minus GLI Race Specific)** |
| FEV_1_ z-scores | –1.32 (1.41) | –1.00 (1.39) | +0.31 (0.22)  [0.31, 0.32] |
| FVC z-scores | –0.87 (1.32) | –0.53 (1.30) | +0.35 (0.22)  [0.34, 0.35] |
| FEV_1_/FVC z-scores | –0.92 (1.37) | –0.98 (1.31) | –0.05 (0.13)  [–0.06, –0.05] |
|  | **Hispanics (n=2375)** | |  |
|  | **NHANES Race Specific reference equations** | **GLI Global reference equations** | **Difference**  **(GLI Global minus GLI Race Specific)** |
| FEV_1_ z-scores | –1.33(1.64) | –0.75 (1.41) | 0.58 (0.38)  [0.57, 0.60] |
| FVC z-scores | –0.76 (1.67) | –0.52 (1.40) | 0.25 (0.65)  [0.22, 0.27] |
| FEV_1_/FVC z-scores | –0.47 (1.70) | –0.50 (1.18) | –0.03 (0.62)  [–0.05, 0.00] |

The differences in the z-scores between reference equations are all statistically significant at *p* < 0.001 (two-sided) except for the FEV_1_/FVC z-scores in the Hispanics, where the *p*-values = 0.028 (two-sided)

**Table S2**. Best fit equations for FEV_1_ (L) and FVC (L) in Blacks and Whites.

| Model 1  FEV_1_ (L)  (n = 2460 Whites)  (n=367 Blacks) | Unstandardized B  Estimate  [95%CI] | R^2^ Contribution | Standardized Coefficients | Standard Error of B  Estimate | *p-*value | Variance Inflation Factor  [95% CI] |
| --- | --- | --- | --- | --- | --- | --- |
| Height^2^ (cm) | 0.0001  [0.0001, 0.0001] | 25% | 0.34 | 0.0000 | <0.001 | 2.00  [1.90, 2.12] |
| Age (years) | –0.0224  [–0.026, –0.018] | 20% | –0.39 | 0.002 | <0.001 | 32.52  [30.25, 34.97] |
| Age^2^ (years) | –0.0001  [–0.0001, 0.000] | 20% | –0.12 | 0.0000 | 0.001 | 32.76  [30.47, 35.22] |
| Sex  (0 = Female, 1 = male) | 0.443  [0.408, 0.478] | 16% | 0.22 | 0.023 | <0.001 | 1.97  [1.87, 2.09] |
| Height^2^ x Race  (0 = White, 1 = Black) | –0.000014  [–0.0000, –0.0000] | 2% | –0.14 | 0.0000 | <0.001 | 1.01  [1.00, 1.71] |
|  |  |  |  |  |  |  |
| Intercept | 1.333  [1.165, 1.501] | -- | -- | 0.086 | <0.001 | -- |
| Model 2  FVC (L)  (n = 2460 Whites)  (n=367 Blacks) | **Unstandardized B**  **Estimate**  **[95%CI]** | **R^2^ Contribution** | **Standardized Coefficients** | **Standard Error of B**  **Estimate** | ***p-*value** | **Variance Inflation Factor**  **[95% CI]** |
| Height^2^ (cm) | 0.0001  [0.001, 0.001] | 31% | 0.49 | 0.0000 | <0.001 | 2.00  [1.90, 2.12] |
| Sex  (0 = Female, 1 = male) | 0.637  [0.593, 0.682] | 22% | 0.32 | 0.0228 | <0.001 | 1.97  [1.87, 2.09] |
| Age (years) | –0.0144  [– 0.020, –0.009] | 13% | –0.25 | 0.0026 | <0.001 | 32.52  [30.25, 34.97] |
| Age^2^ (years) | –0.00014  [–0.0001, 0.0000] | 13% | –0.26 | 0.0000 | 0.123 | 32.76  [30.47, 35.22] |
| Height^2^ x Race  (0 = White, 1 = Black) | –0.00002  [–0.0000, –0.0000] | 3% | –0.20 | 0.0000 | <0.001 | 1.01  [1.00, 1.71] |
| Intercept | 0.711  [0.496, 0.926] | -- | -- | 0.11 | <0.001 | -- |

. For FEV_1_: BIC = 1885, Adjusted R^2^ = 0.84, Residual Standard Error = 0.335. *F*(5,2821) = 2901, *p <* 0.001. Durbin-Watson = 1.87. For FVC: BIC = 3280, Adjusted R^2^ = 0.83, Residual Standard Error = 0.43. *F*(5, 2821) = 2732, *p <* 0.001. Durbin-Watson = 1.91. The standardized regression coefficient represents how many standard deviations a dependent variable will change per one standard deviation increase in the predictor variable. Blacks have a lower FEV_1_ and FVC compared to whites.

**Table S3**. Best fit equations for FEV_1_ (L) and FVC in Hispanics and Whites.

| Model 3  FEV_1_ (L); (n = 2460 Whites)  (n=944 Hispanics) | Unstandardized B  Estimate  [95%CI] | R^2^ contribution | Standardized Coefficients | SE | *p-*value | Variance Inflation Factor [95% CI] |
| --- | --- | --- | --- | --- | --- | --- |
| Height^2^ (cm) | 0.0001  [0.0001, 0.0001] | 24% | 0.36 | 0.000 | <0.001 | 2.4  [2.3, 2.5] |
| Age (years) | 0.0212  [0.0246 –0.0179] | 21% | –0.38 | 0.002 | <0.000 | 30.7  [28.8, 32.8] |
| Age^2^ | –0.0001  [–0.0001, 0.0000] | 20% | –0.14 | 0.000 | <0.001 | 30.9  [29.0, 33.0] |
| Sex (0 = Female, 1 = male) | 0.469  [0.438, 0.499] | 16% | 0.23 | 0.016 | <0.001 | 1.9  [1.8, 2.0] |
| Race  (0 = White, 1 = Hispanic) | –0.563  [–0.773, –0.353] | 2% | –0.56 | 0.107 | <0.001 | 73.9  [69.1, 78.9] |
| Height^2^ x Race  (0 = White, 1 = Hispanic) | 0.00001023  [0.0000, 0.0000] | 2% | 0.25 | 0.000 | <0.001 | 71.7  [67.1, 76.7] |
| Intercept | 1.318  [1.162, 1.474] | -- | -- | 0.080 | <0.001 | -- |
| Model 4  FVC (L); (n = 2460 Whites)  (n=944 Hispanics) | **Unstandardized B**  **Estimate**  **[95%CI]** | **R^2^ contribution** | **Standardized Coefficients** | **SE** | ***p-*value** | **Variance Inflation Factor [95% CI]** |
| Height^2^ (cm) | 0.00015  [0.001, 0.002] | 28% | 0.52 | 0.000 | <0.001 | 3.2  [3.0, 3.4] |
| Sex (0 = Female, 1 = male) | 0.665  [0.627, 0.704] | 19% | 0.32 | 0.020 | <0.001 | 1.9  [1.8, 2.0] |
| Age^2^ (years) | –0.0002  [–0.0002, –0.0001] | 13% | –0.32 | 0.000 | <0.001 | 32.2  [30.1, 34.4] |
| Age (years) | –0.011  [– 0.016, –0.007] | 13% | –0.20 | 0.002 | <0.001 | 32.3  [30.2, 34.5] |
| Weight (kg) | –0.0023  [ –0.0035, –0.001] | 7% | –0.002 | 0.001 | 0.0004 | 1.9  [1.8, 2.0] |
| Race  (0 = White, 1 = Hispanic) | –0.705  [–0.971, –0.439] | 2% | –0.70 | 0.014 | <0.001 | 73.9  [69.1, 78.9] |
| Height^2^ x Race  (0 = White, 1 = Hispanic) | 0.00002323  [0.000, 0.0000] | 1% | 0.28 | 0.000 | <0.001 | 71.8  [67.2, 76.7] |
| Intercept | 0.5872  [0.3876, 0.7867] | -- | -- | 0.102 | <0.001 | -- |

SE = standard error. For FEV_1_: BIC = 2079, Adjusted R^2^ = 085, Residual Standard Error = 0.326. *F*(6, 3397) = 2079, *p <* 0.0001. Durbin-Watson = 1.91. For FVC: BIC = 3693, Adjusted R^2^ = 0.84, Residual Standard Error = 0.412. *F*(7, 3396) = 2576, *p <* 0.0001. Durbin-Watson = 1.95. The standardized regression coefficient represents how many standard deviations a dependent variable will change per one standard deviation increase in the predictor variable. Hispanics have smaller FEV_1_ than Whites by ~560 mL (0.56 L). Hispanics have a smaller FVC by ~180 mL (0.18 L) at a height of 150 cm, and by 175 cm, the ethnic difference disappears.

**Table S4**. Best fit equations for FEV_1_/FVC ratio.

| Model 5  FEV_1_/FVC  Blacks & Whites  (n = 2460 Whites)  (n=367 Blacks) | Unstandardized B  Estimate  [95%CI] | R^2^ contribution | Standardized Coefficients | SE | *p-*value | Variance Inflation Factor [95% CI] |
| --- | --- | --- | --- | --- | --- | --- |
| Age (years) | –0.0018  [– 0.0019, – 0.0017] | 27% | –0.032 | 0.000 | <0.001 | 1.0  [1.0, 1.1] |
| Sex  (0 = Female, 1 = male) | –0.0124  [–0.018, –0.007] | 3% | –0.006 | 0.0030 | <0.001 | 2.0  [1.9, 2.1] |
| Height (cm) | –0.0008  [–0.0011, –0.0006] | 2% | –0.001 | 0.0001 | <0.001 | 2.0  [1.9, 2.1] |
| Race  (0=White, 1 = Black) | 0.0142  [0.009, 0.020] | 1% | 0.014 | 0.0030 | <0.001 | 1.0  [1.0, 2.2] |
| Intercept | 1.017  [0.974, 1.060] | -- | -- | 0.0200 | <0.001 | -- |
| Model 6  FEV_1_/FVC  Hispanics & Whites  (n = 2460 Whites)  (n=944 Hispanics) | **Unstandardized B**  **Estimate**  **[95%CI]** | **R^2^ contribution** | **Standardized Coefficients** | **SE** | ***p-*value** | **Variance Inflation Factor**  **[95% CI]** |
| Age (years) | –0.0018  [– 0.0019, – 0.0017] | 27% | –0.03 | 0.000 | <0.001 | 1.1  [1.1, 1.1] |
| Race  (0 = White, 1 = Hispanic) | 0.0171  [0.013, 0.0212] | 4% | 0.0008 | 0.0030 | <0.001 | 1.2  [1.2, 1.3] |
| Height (cm) | –0.0009  [–0.0012, –0.0007] | 3% | –0.009 | 0.0001 | <0.001 | 2.1  [2.0, 2.2] |
| Sex  (0 = Female, 1 = Male) | –0.0128  [–0.017, –0.008] | 3% | –0.006 | 0.0030 | <0.001 | 1.9  [1.8, 2.0] |
| Intercept | 1.036  [0.997, 1.076] | -- | -- | 0.0200 | <0.001 | -- |

SE = standard error. For Model 5, the BIC = –8872, Adjusted R^2^ = 0.33, Residual Standard Error = 0.050. *F*(4,2822) = 344.4, *p <* 0.001. Durbin-Watson = 1.95. For Model 6, the BIC = –10762, Adjusted R^2^ = 0.37, Residual Standard Error = 0.0495. *F*(4,3399) = 489.2, *p <* 0.001. Durbin-Watson = 1.97. The standardized regression coefficient represents how many standard deviations a dependent variable will change per one standard deviation increase in the predictor variable.

**Table S5.**  Prediction Accuracy (RMSE) for Race-Specific and Race-Neutral models using repeated *k*-fold cross validation

|  | BIC | Median RMSE | Minimum RMSE | Maximum RMSE | 95% CI of the RMSE |
| --- | --- | --- | --- | --- | --- |
| Model 1, FEV_1_ (L) |  |  |  |  |  |
| Race-Specific (Blacks) | 1886 | 0.338***** | 0.314 | 0.353 | 0.317 to 0.350 |
| Race-Neutral | 2302 | 0.363 | 0.324 | 0.386 | 0.330 to 0.383 |
|  |  |  |  |  |  |
| Model 2, FVC (L) |  |  |  |  |  |
| Race-Specific (Blacks) | 3280 | 0.424***** | 0.398 | 0.458 | 0.399 to 0.457 |
| Race-Neutral | 3808 | 0.462 | 0.437 | 0.516 | 0.438 to 0.512 |
|  |  |  |  |  |  |
| Model 3, FEV_1_ (L) |  |  |  |  |  |
| Race-Specific (Hispanics) | 2079 | 0.326 | 0.308 | 0.343 | 0.310 to 0.343 |
| Race-Neutral | 2090 | 0.326 | 0.308 | 0.342 | 0.310 to 0.342 |
|  |  |  |  |  |  |
| Model 4, FVC(L) |  |  |  |  |  |
| Race-Specific (Hispanics) | 3693 | 0.416 | 0.387 | 0.435 | 0.389 to 0.434 |
| Race-Neutral | 3721 | 0.417 | 0.388 | 0.438 | 0.391 to 0.436 |
|  |  |  |  |  |  |
| Model 5, FEV_1_/FVC |  |  |  |  |  |
| Race-Specific (Blacks) | –8872 | 0.050 | 0.048 | 0.052 | 0.048 to 0.052 |
| Race-Neutral | –8848 | 0.050 | 0.048 | 0.052 | 0.048 to 0.052 |
| Model 6, FEV_1_/FVC |  |  |  |  |  |
| Race-Specific (Hispanics) | –10762 | 0.049 | 0.047 | 0.052 | 0.047 to 0.052 |
| Race-Neutral | –10695 | 0.050 | 0.047 | 0.053 | 0.047 to 0.053 |
|  |  |  |  |  |  |

*****The RMSE (Root Mean Square Error) is lower when using race-specific reference equations for Blacks and Whites compared to race-neutral equations for FEV_1_ and FVC after permutation (p < 0.01). For all other race-specific reference equations, the RMSE was similar to race-neutral equations, with no significant differences. There were 367 Blacks, 2460 Whites, and 944 Hispanics for model development.

**Table S6.**  The correlation coefficients between actual and predicted values from repeated *k*-fold cross validation.

|  | BIC | Median Correlation Coefficient | Minimum Correlation Coefficient | Maximum correlation coefficient | 95% CI of the correlation coefficient |
| --- | --- | --- | --- | --- | --- |
| Model 1, FEV_1_ (L) |  |  |  |  |  |
| Race-Specific (Blacks) | 1886 | 0.913***** | 0.904 | 0.930 | 0.905 to 0.929 |
| Race-Neutral | 2302 | 0.901 | 0.878 | 0.926 | 0.88 to 0.923 |
|  |  |  |  |  |  |
| Model 2, FVC (L) |  |  |  |  |  |
| Race-Specific (Blacks) | 3280 | 0.911***** | 0.897 | 0.921 | 0.899 to 0.920 |
| Race-Neutral | 3808 | 0.892 | 0.866 | 0.904 | 0.870 to 0.903 |
|  |  |  |  |  |  |
| Model 3, FEV_1_ (L) |  |  |  |  |  |
| Race-Specific (Hispanics) | 2079 | 0.921 | 0.907 | 0.927 | 0.909 to 0.927 |
| Race-Neutral | 2090 | 0.921 | 0.907 | 0.927 | 0.909 to 0.927 |
|  |  |  |  |  |  |
| Model 4, FVC(L) |  |  |  |  |  |
| Race-Specific (Hispanics) | 3693 | 0.917 | 0.902 | 0.925 | 0.905 to 0.924 |
| Race-Neutral | 3721 | 0.917 | 0.903 | 0.924 | 0.905 to 0.923 |
|  |  |  |  |  |  |
| Model 5, FEV_1_/FVC |  |  |  |  |  |
| Race-Specific (Blacks) | –8872 | 0.582 | 0.493 | 0.639 | 0.499 to 0.633 |
| Race-Neutral | –8848 | 0.571 | 0.482 | 0.635 | 0.491 to 0.630 |
|  |  |  |  |  |  |
| Model 6, FEV_1_/FVC |  |  |  |  |  |
| Race-Specific (Hispanics) | –10762 | 0.600 | 0.562 | 0.658 | 0.564 to 0.653 |
| Race-Neutral | –10695 | 0.589 | 0.546 | 0.656 | 0.550 to 0.650 |
|  |  |  |  |  |  |

*****The correlation between actual and predicted values in the test set was higher when using Race-Specific reference equations compared to Race-Neutral equations for FEV_1_ and FVC after permutation (*p* < 0.01). All other models showed similar correlation coefficients and were not significant. There were 367 Blacks, 2460 Whites, and 944 Hispanics for model development.

**Figure S1**. Top panel: The number of subjects per race/ethnicity and sex used for developing reference equations. These subjects had normal spirometry. Top panel: sex distribution by ethnicity/race. Bottom panel: age distribution by sex.


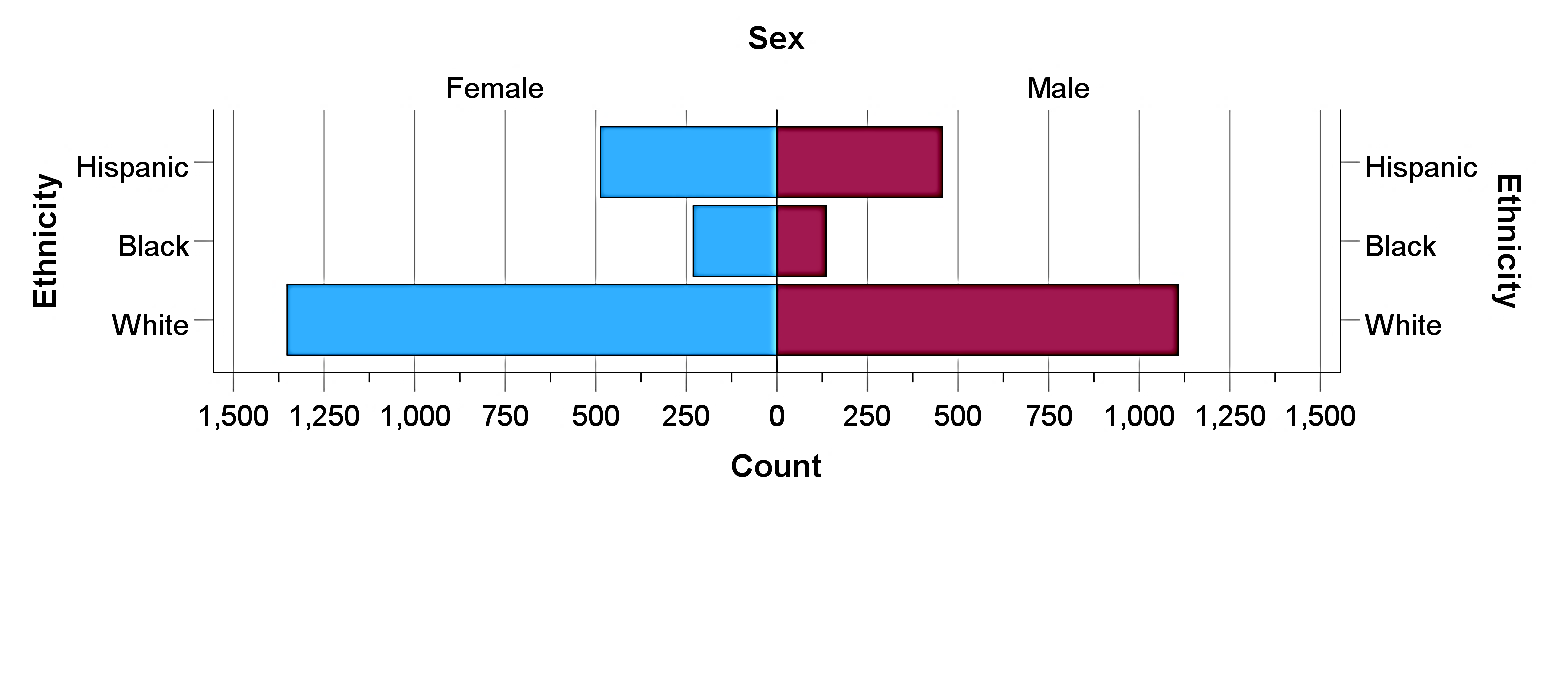


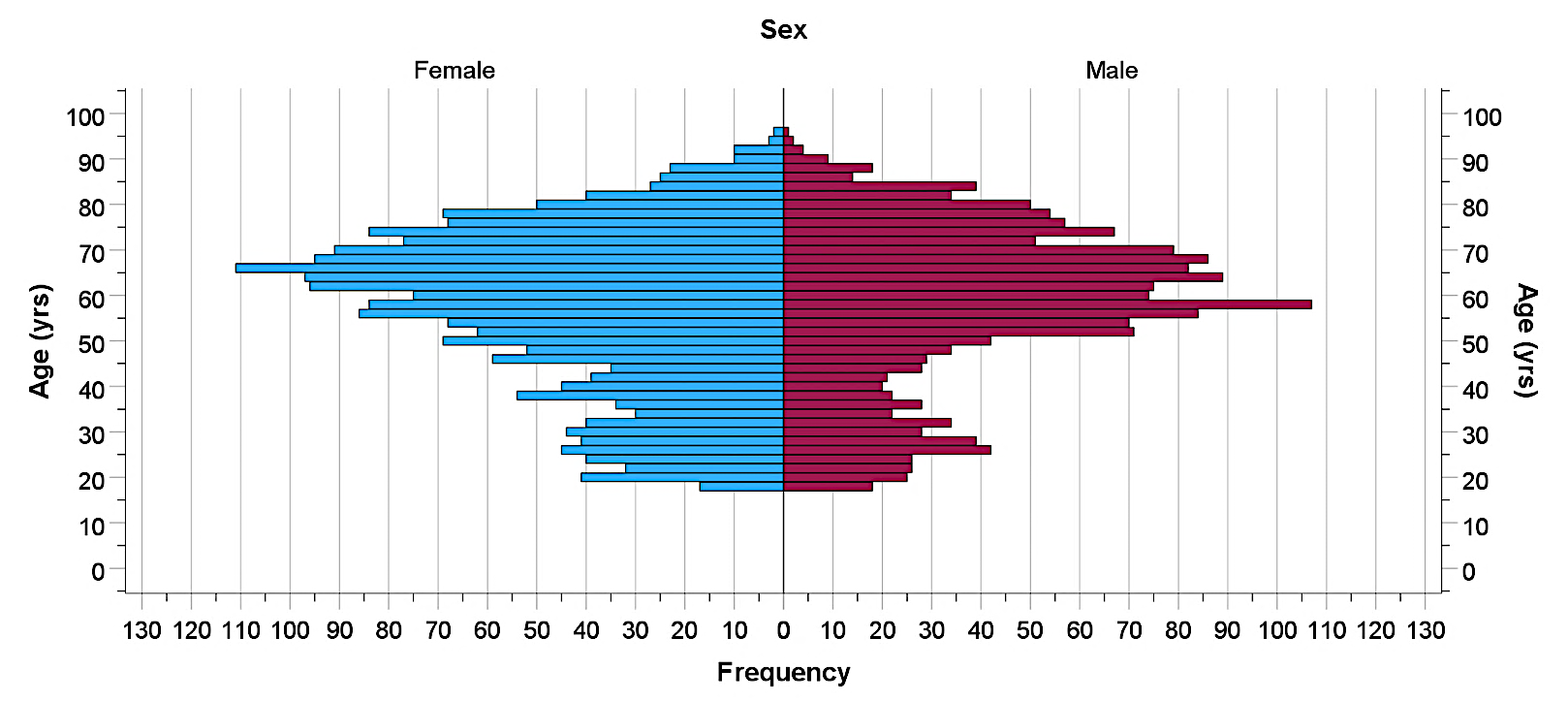


**Figure S2.** Visual Check of various linear regression model assumptions in Model 1. The *performance* package in R created these visual plots. Ethnicity: 0 = White; 1 = Black. Sex: 0 = female, 1 = male. The high **VIFs for** Age and Age^2^, and Height^2^ x Ethnicity **are due to their powers and products. These** are said to be one quadratic effect and not separate effects, so they can be ignored.


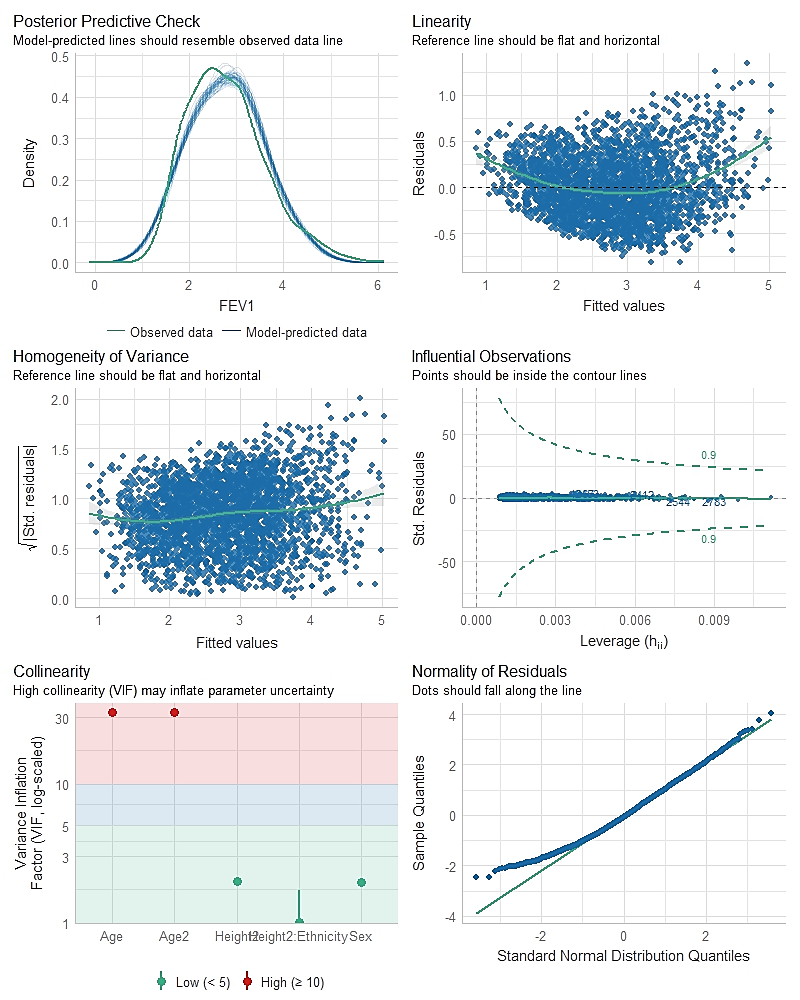


**Height^2^ x Ethnicity**

**Sex**

**Height^2^**

**Age^2^**

**Age**

**Figure S3.** Visual Check of various linear regression model assumptions in Model 2. The *performance* package in R created these visual plots. Ethnicity: 0 = White; 1 = Black. Sex: 0 = female, 1 = male. The high **VIFs for** Age and Age^2^, and Height^2^ x Ethnicity **are due to their powers and products. These** are said to be one quadratic effect and not separate effects, so they can be ignored.


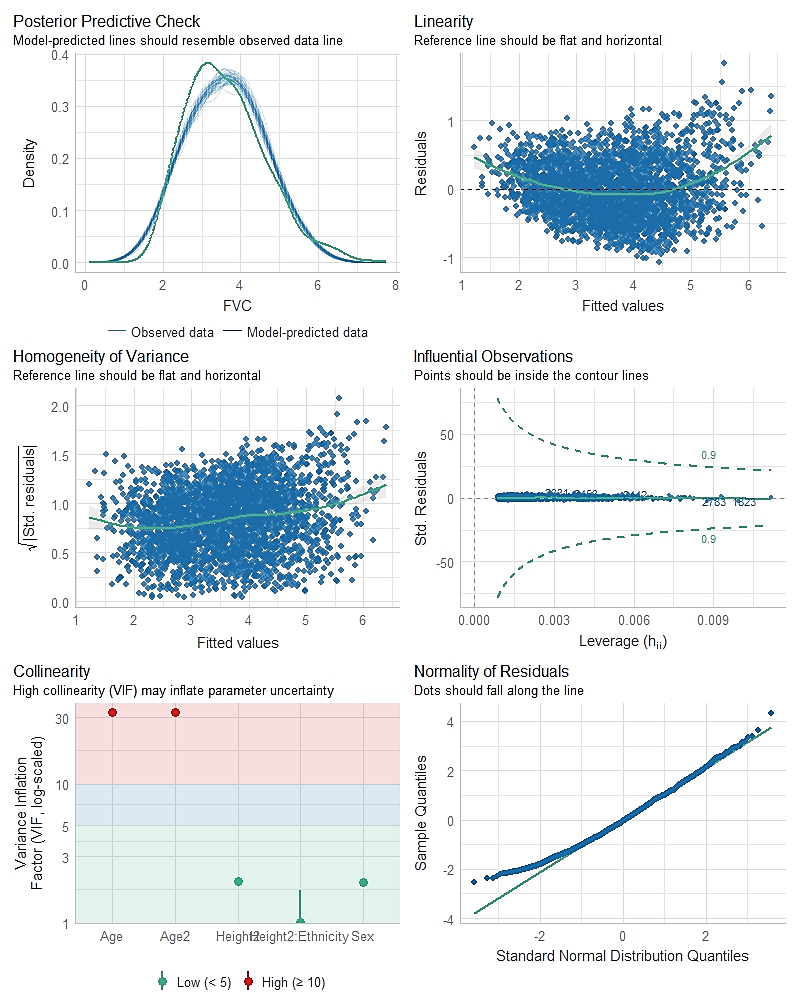


**Sex**

**Height**

**Height^2^ x Ethnicity**

**Age^2^**

**Age**

**Figure S4.** Visual Check of various linear regression model assumptions in Model 3. The *performance* package in R created these visual plots. Ethnicity: 0 = White; 1 = Hispanic. Sex: 0 = female, 1 = male. The high **VIFs for** Age and Age^2^, and Height^2^ x Ethnicity **are due to their powers and products. These** are said to be one quadratic effect and not separate effects, so they can be ignored.


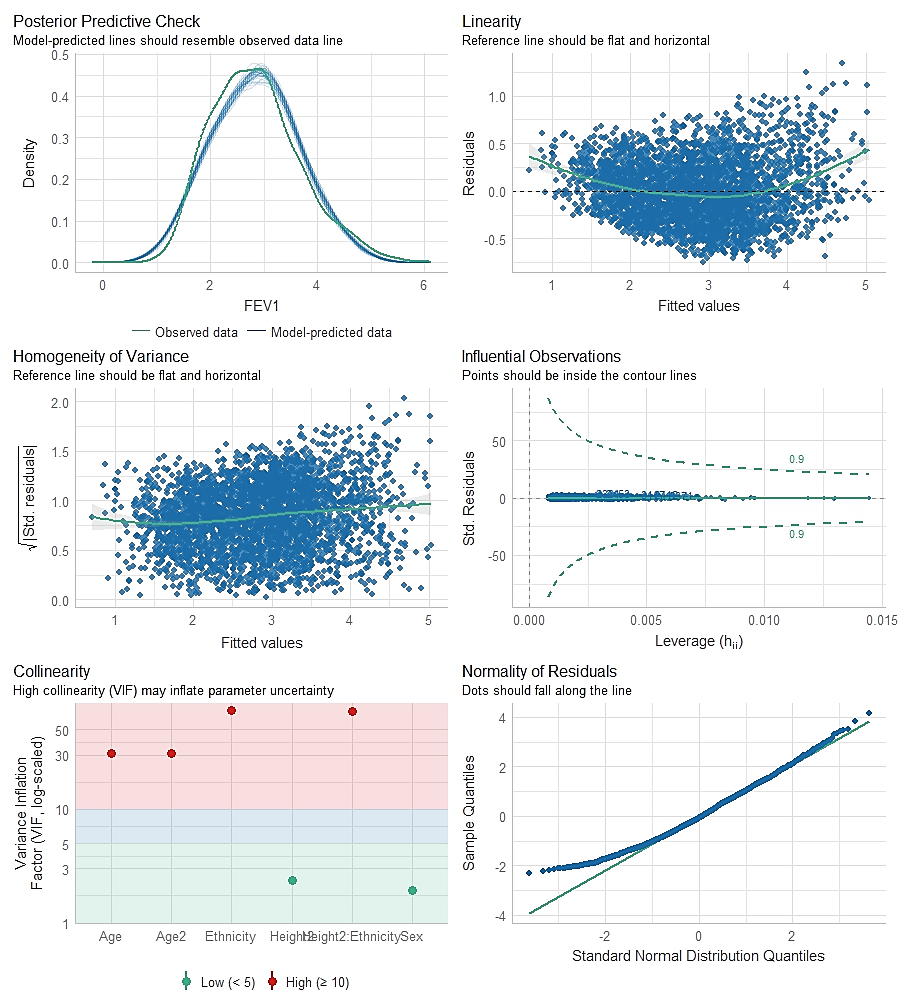


**Height**

**Sex**

**Height^2^ x Ethnicity**

**Ethnicity**

**Age^2^**

**Age**

**Figure S5.** Visual Check of various linear regression model assumptions in Model 4. The *performance* package in R created these visual plots. Ethnicity: 0 = White; 1 = Hispanic. Sex: 0 = female, 1 = male. The high **VIFs for** Age and Age^2^, and Height^2^ x Ethnicity **are due to their powers and products. These** are said to be one quadratic effect and not separate effects, so they can be ignored.


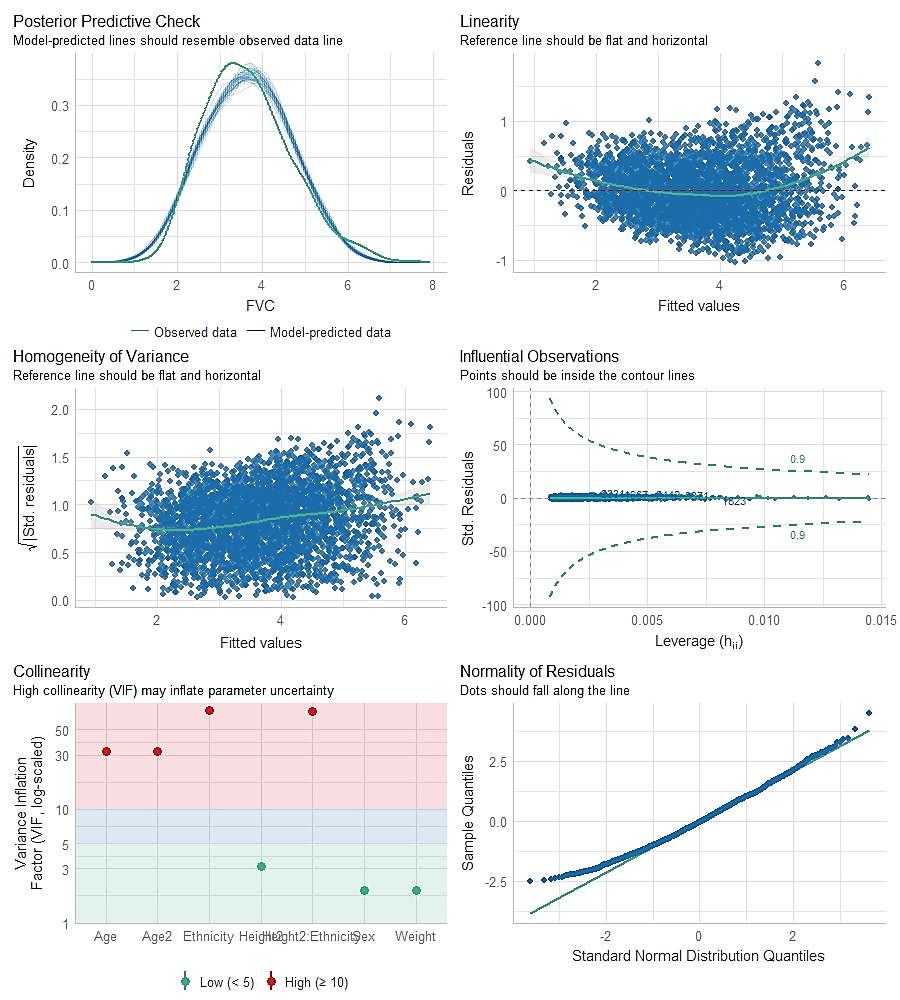


**Height^2^ x Ethnicity**

**Sex**

**Weight**

**Height**

**Ethnicity**

**Age**

**Age^2^**

**Figure S6.** Visual Check of various regression model assumptions in Model 5. The *performance* package in R created these visual plots. Ethnicity: 0 = White; 1 =Black. Sex: 0 = female, 1 = male.


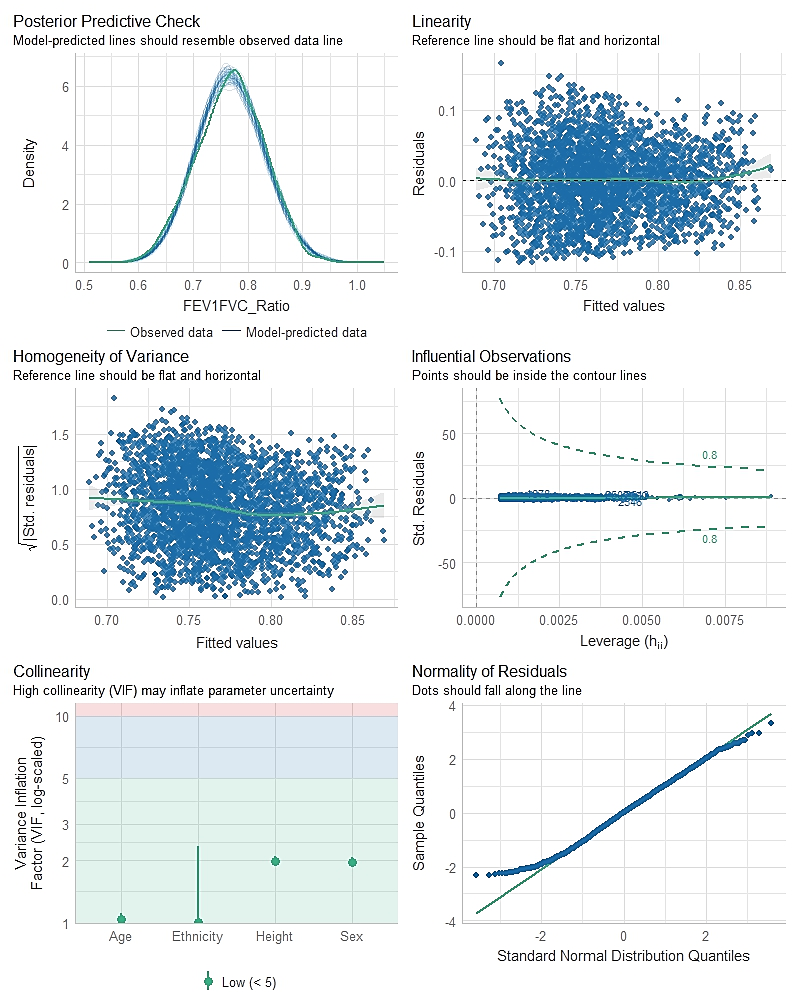


**Figure S7.** Visual Check of various regression model assumptions in Model 6. The *performance* package in R created these visual plots. Ethnicity: 0 = White; 1 = Hispanic. Sex: 0 = female, 1 = male.


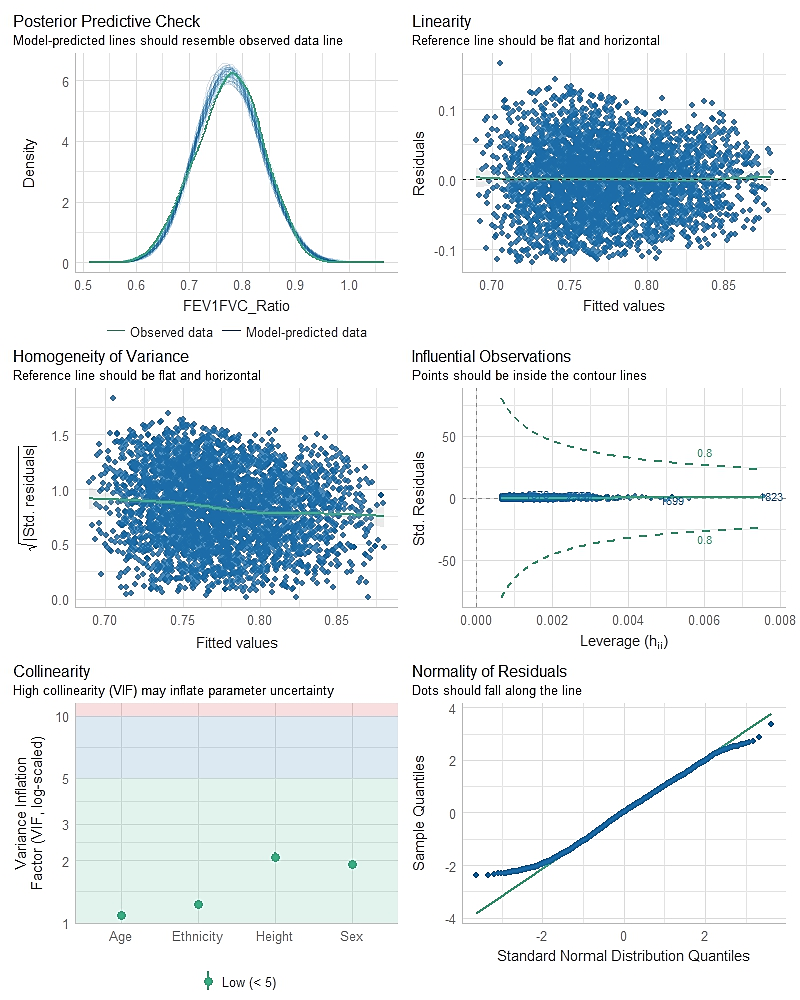


**Figure S8**. Density plot of Root Mean Square Error (RMSE) values between Race-Specific and Race-Neutral reference equations for FEV_1_/FVC Ratio, (Blacks vs Whites).

Race-Specific Reference Equation (**Blue Curve**): This equation includes race as a covariate, with race coded as 0 for Mexican and 1 for White. The Race-Neutral Reference Equation (**Green Curve**) does not include race as a covariate and treats all subjects as one race.

**Left panel:** The density plot shows that the Race-Specific reference equation tends to have similar RMSE values compared to the Race-Neutral reference equation, as both blue and green curves completely overlap. The peak density for the Race-Specific reference equation is similar to the Race-Neutral reference equation, suggesting similar performance. Permutation test results: RMSE Difference = 0.0002, two-sided *p*-value = 0.9556. Thus, the similarities between both equations are unlikely to have occurred by chance. The bootstrapped test results also did not show a statistically significant difference between the equations (two-sided, *p* = 0.7782),which suggests that in real-world applications, the choice between race-specific and race-neutral reference equations may not be so important for the FEV_1_/FVC Ratio when comparing Blacks vs Whites.

**Right panel:** The blue line (Race-Specific reference equation for the FEV_1_/FVC Ratio) is overlapping the green line (Race-Neutral reference equation for the FEV_1_/FVC Ratio), indicating that the Race-Specific reference equation generally has similar correlation coefficients (permutation test results, correlation difference = –0.011, two-sided p-value = 0.685). The bootstrap results did not show a statistically significant difference between the two equations (*p* = 0.698), which implies that there is no practical difference between them.


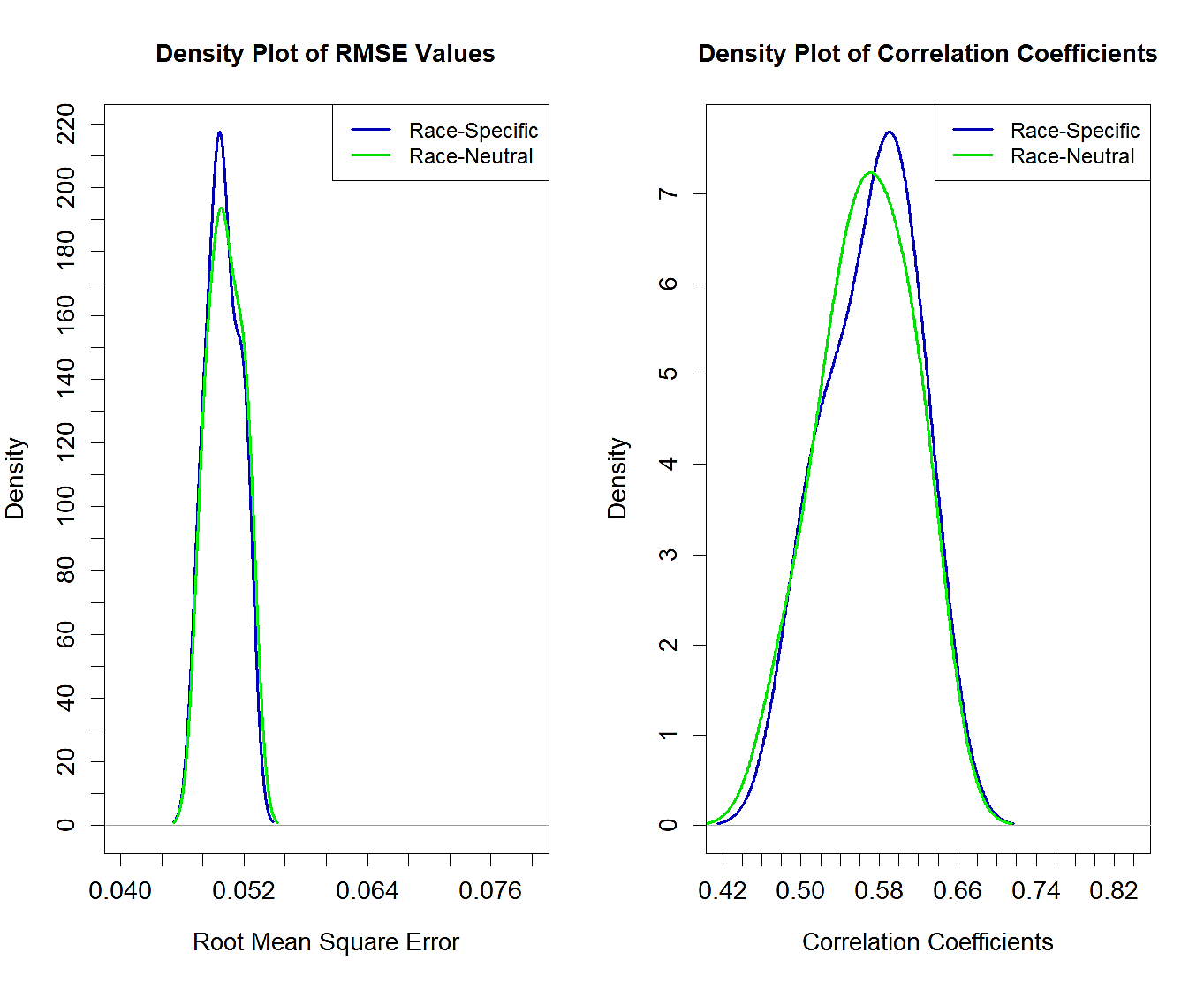


**Figure S9.** Density plot of Root Mean Square Error (RMSE) values between Race-Specific and Race-Neutral reference equations for FEV_1_, (Hispanics vs Whites).

Race-Specific Reference Equation (**Blue Curve**): This equation includes race as a covariate, with race coded as 0 for Mexican and 1 for White. The Race-Neutral Reference Equation (**Green Curve**) does not include race as a covariate and treats all subjects as one race.

**Left panel:** The density plot shows that the Race-Specific reference equation tends to have similar RMSE values compared to the Race-Neutral reference equation, as both blue and green curves completely overlap. The peak density for the Race-Specific reference equation is similar to the Race-Neutral reference equation, suggesting similar performance. Permutation test results: RMSE Difference = 0.000 L, two-sided *p*-value = 0.951. Thus, the similarities between both equations are unlikely to have occurred by chance. The bootstrapped test results also did not show a statistically significant difference between the equations (two-sided, *p* = 0.983),which suggests that in real-world applications, the choice between race-specific and race-neutral reference equations may not be so important for FEV_1_ when comparing Hispanics vs Whites.

**Right panel:** The blue line (Race-Specific reference equation) is overlapping the green line (Race-Neutral reference equation), indicating that the Race-Specific equation generally has similar correlation coefficients (permutation test results, correlation difference = 0.0001, two-sided *p*-value = 1.00). The bootstrap results did not show a statistically significant difference between the two equations (*p* = 0.973), which implies that there is no practical difference between them.


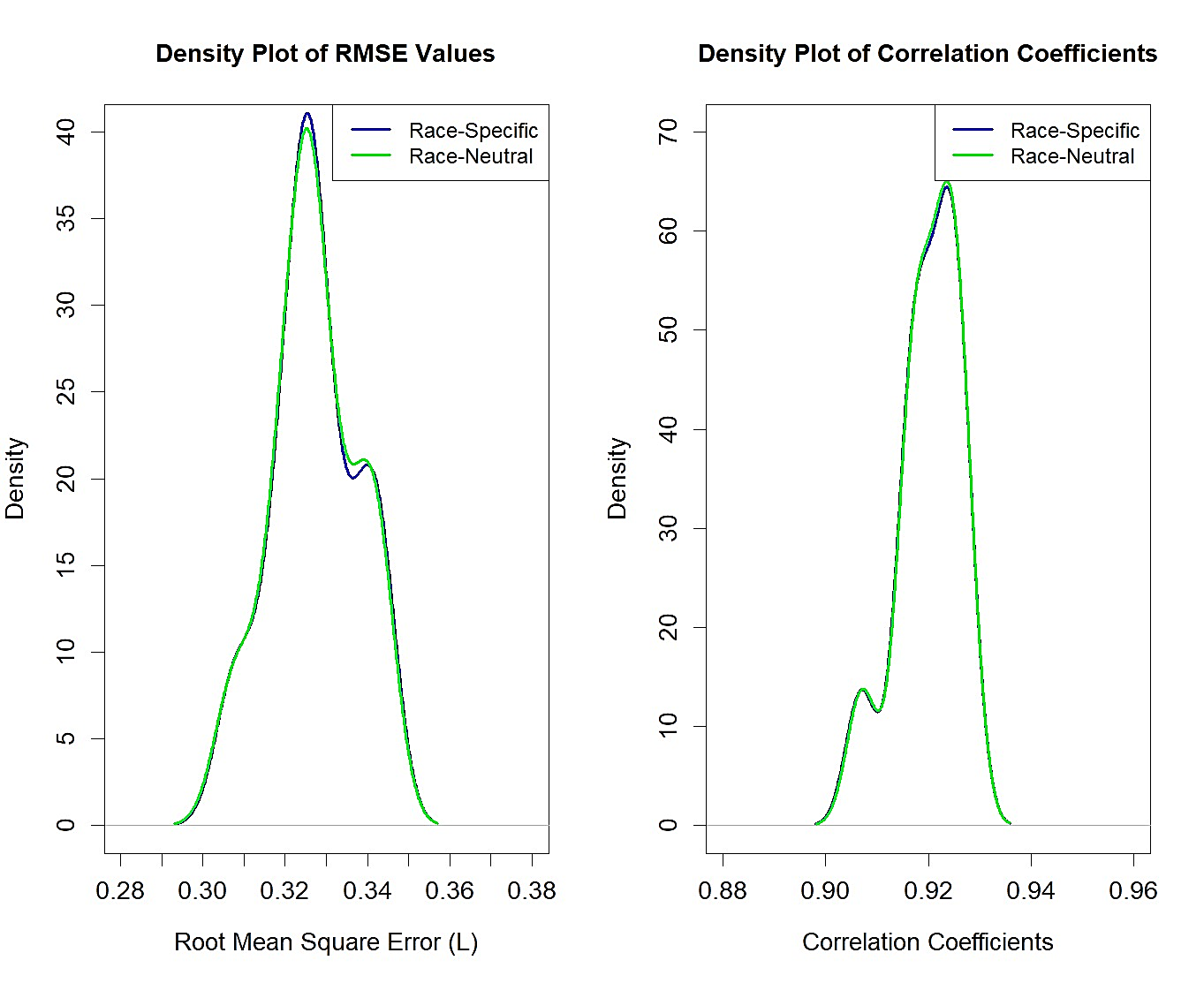


**Figure S10**. Density plot of Root Mean Square Error (RMSE) values between Race-Specific and Race-Neutral reference equations for FVC, (Hispanics vs Whites).

Race-Specific Reference Equation (**Blue Curve**): This equation includes race as a covariate, with race coded as 0 for Mexican and 1 for White. The Race-Neutral Reference Equation (**Green Curve**) does not include race as a covariate and treats all subjects as one race.

**Left panel**: The density plot shows that the Race-Specific reference equation tends to have similar RMSE values compared to the Race-Neutral reference equation, as both blue and green curves completely overlap. The peak density for the Race-Specific reference equation is similar to the Race-Neutral reference equation, suggesting similar performance. Permutation test results: RMSE Difference = 0.015 L two-sided *p*-value = 0.965. Thus, the similarities between both equations are unlikely to have occurred by chance. The bootstrapped test results also did not show a statistically significant difference between the equations (two-sided, *p* = 0.901),which suggests that in real-world applications, the choice between Race-Specific and Race-Neutral reference equations may not be so important for FVC when comparing Hispanics vs Whites.

**Right panel:** The blue line (Race-Specific Reference Equation) is overlapping the green line (Race-Neutral Reference Equation), indicating that the Race-Specific model generally has similar correlation coefficients (permutation test results, correlation difference = –0.002, two-sided *p*-value = 0.762). The bootstrap results did not show a statistically significant difference between the two equations (*p* = 0.464), which implies that there is no practical difference between them.


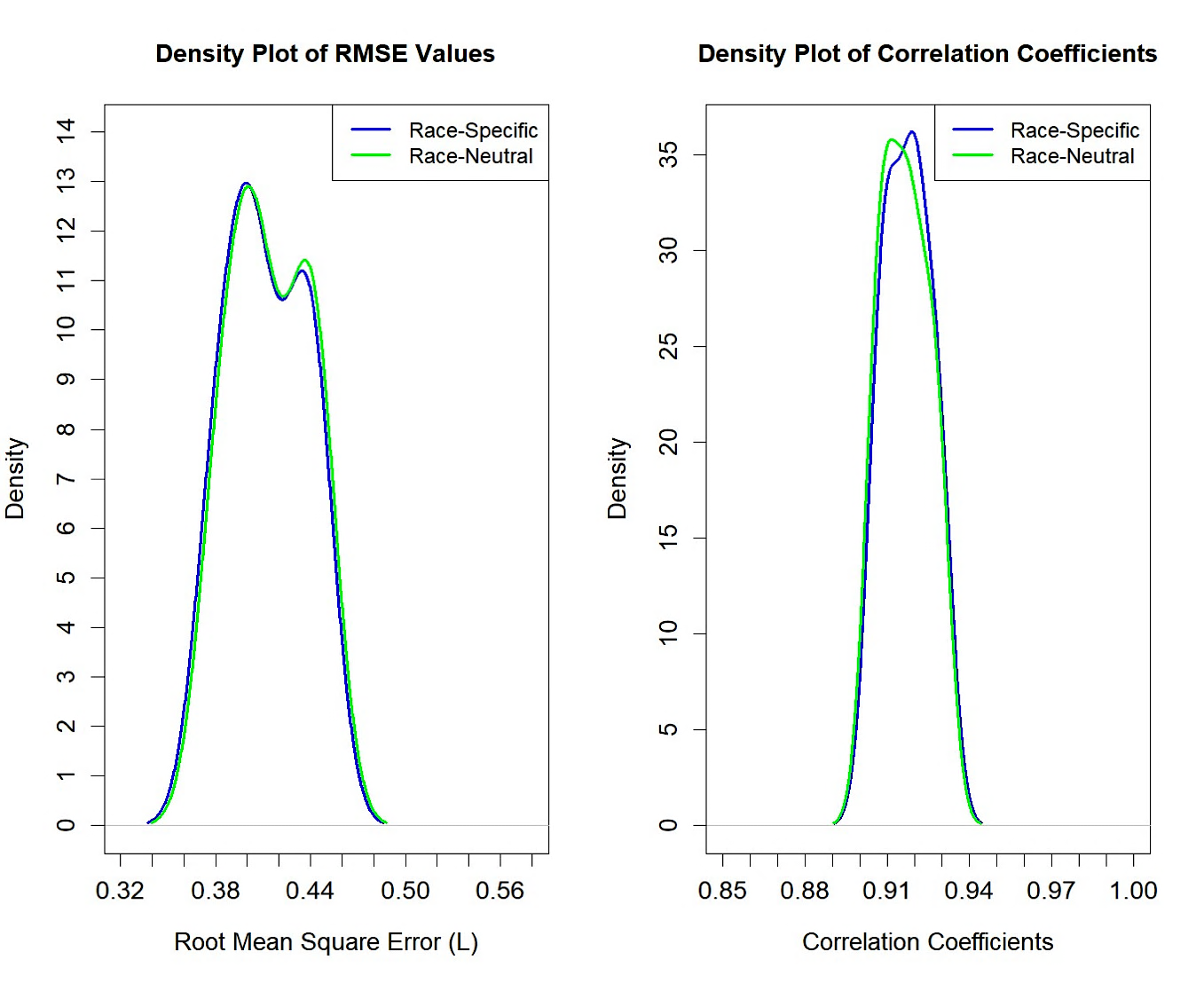


**Figure S11**. Density plot of Root Mean Square Error (RMSE) values between Race-Specific and Race-Neutral reference equations for the FEV_1_/FVC ratio (Hispanics vs Whites).

Race-Specific Reference Equation (**Blue Curve**): This model includes race as a covariate, with race coded as 0 for Mexican and 1 for White. The Race-Neutral Reference Equation (**Green Curve**) does not include race as a covariate and treats all subjects as one race.

**Left panel:** The density plot shows that the Race-Specific equation tends to have similar RMSE values compared to the Race-Neutral reference equation, as both blue and green curves completely overlap. The peak density for the Race-Specific reference equation is similar to the Race-Neutral reference equation, suggesting similar performance. Permutation test results: RMSE Difference = 0.0003, two-sided *p*-value = 0.826. Thus, the similarities between both equations are unlikely to have occurred by chance. The bootstrapped test results also did not show a statistically significant difference between the equations (two-sided, *p* = 0.815), which suggests that in real-world applications, the choice between race-specific and race-neutral reference equations may not be so important for the FEV_1_/FVC ratio when comparing Hispanics vs Whites.

**Right panel:** The blue line (Race-Specific reference equation) is overlapping the green line (Race-Neutral reference equation), indicating that the Race-Specific equation generally has similar correlation coefficients (permutation test results, correlation difference = –0.011, two-sided *p*-value = 0.762). The bootstrap results did not show a statistically significant difference between the two equations (*p* = 0.692), which implies that there is no practical difference between them.


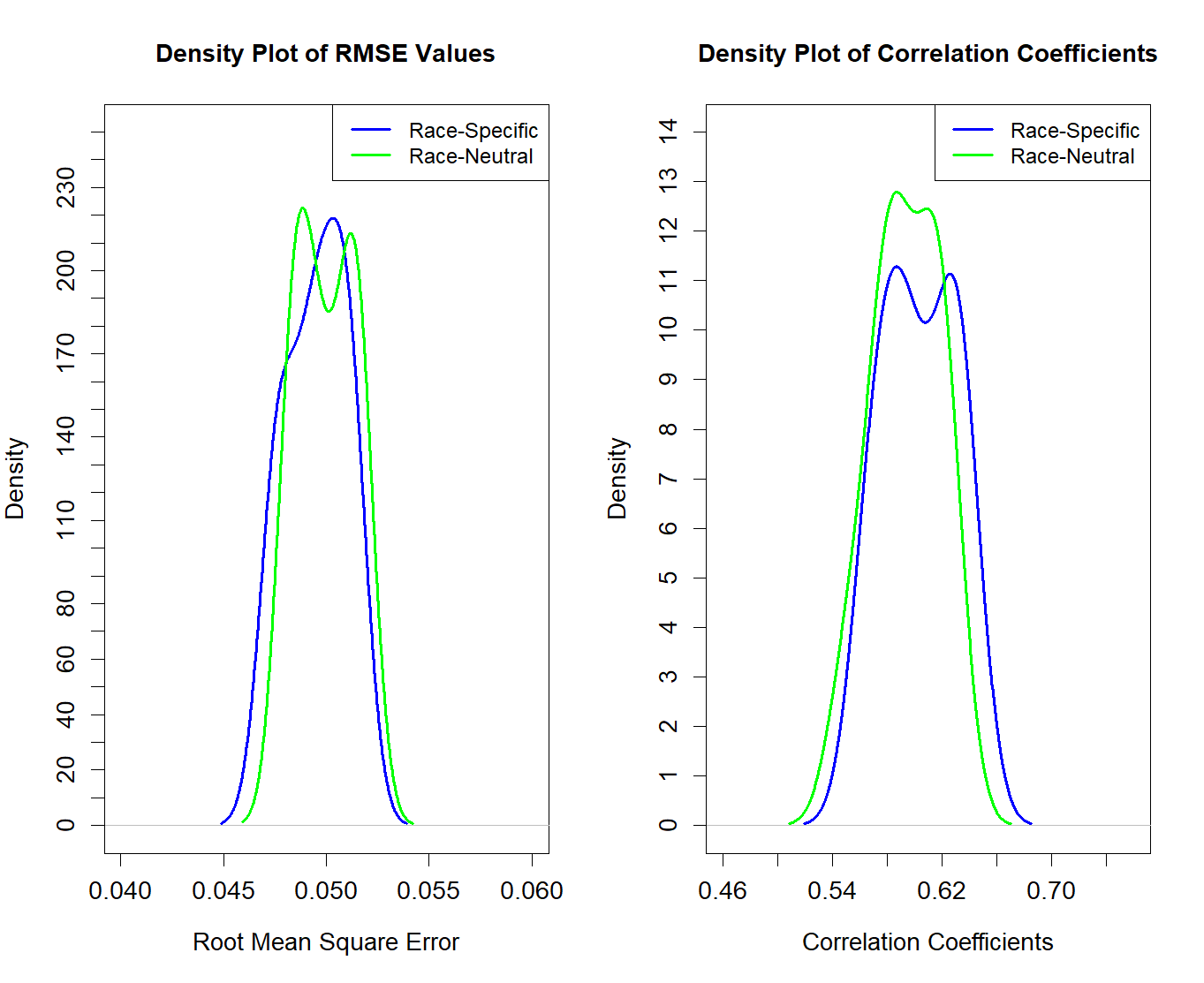

Supplement: Supplementary file 1 — Appendix S1. [file PHY2-13-e70212-s001.docx]
